# PGF_2α_ signaling drives fibrotic remodeling and fibroblast population dynamics in mice

**DOI:** 10.1172/jci.insight.172977

**Published:** 2023-12-22

**Authors:** Luis R. Rodriguez, Soon Yew Tang, Willy Roque Barboza, Aditi Murthy, Yaniv Tomer, Tian-Quan Cai, Swati Iyer, Katrina Chavez, Ujjalkumar Subhash Das, Soumita Ghosh, Charlotte H. Cooper, Thalia T. Dimopoulos, Apoorva Babu, Caitlin Connelly, Garret A. FitzGerald, Michael F. Beers

**Affiliations:** 1 Pulmonary, Allergy, and Critical Care Division, Department of Medicine,; 2 PENN-CHOP Lung Biology Institute, and; 3 Institute for Translational Medicine and Therapeutics, Department of Systems Pharmacology and Translational Therapeutics, Perelman School of Medicine at the University of Pennsylvania, Philadelphia, Pennsylvania, USA.; 4 Calico Life Sciences LLC, South San Francisco, California, USA.

**Keywords:** Pulmonology, Eicosanoids, Fibrosis, Molecular biology

## Abstract

Idiopathic pulmonary fibrosis (IPF) is a chronic parenchymal lung disease characterized by repetitive alveolar cell injury, myofibroblast proliferation, and excessive extracellular matrix deposition for which unmet need persists for effective therapeutics. The bioactive eicosanoid, prostaglandin F_2α_, and its cognate receptor FPr (*Ptgfr*) are implicated as a TGF-β1–independent signaling hub for IPF. To assess this, we leveraged our published murine PF model (I^ER^-*Sftpc^I73T^*) expressing a disease-associated missense mutation in the surfactant protein C (*Sftpc*) gene. Tamoxifen-treated I^ER^-*Sftpc^I73T^* mice developed an early multiphasic alveolitis and transition to spontaneous fibrotic remodeling by 28 days. I^ER^-*Sftpc^I73T^* mice crossed to a *Ptgfr-*null (FPr^–/–^) line showed attenuated weight loss and gene dosage–dependent rescue of mortality compared with FPr^+/+^ cohorts. I^ER^-Sftpc^I73T^/FPr^–/–^ mice also showed reductions in multiple fibrotic endpoints for which administration of nintedanib was not additive. Single-cell RNA-Seq, pseudotime analysis, and in vitro assays demonstrated *Ptgfr* expression predominantly within adventitial fibroblasts, which were reprogrammed to an “inflammatory/transitional” cell state in a PGF_2α_ /FPr-dependent manner. Collectively, the findings provide evidence for a role for PGF_2α_ signaling in IPF, mechanistically identify a susceptible fibroblast subpopulation, and establish a benchmark effect size for disruption of this pathway in mitigating fibrotic lung remodeling.

## Graphical abstract

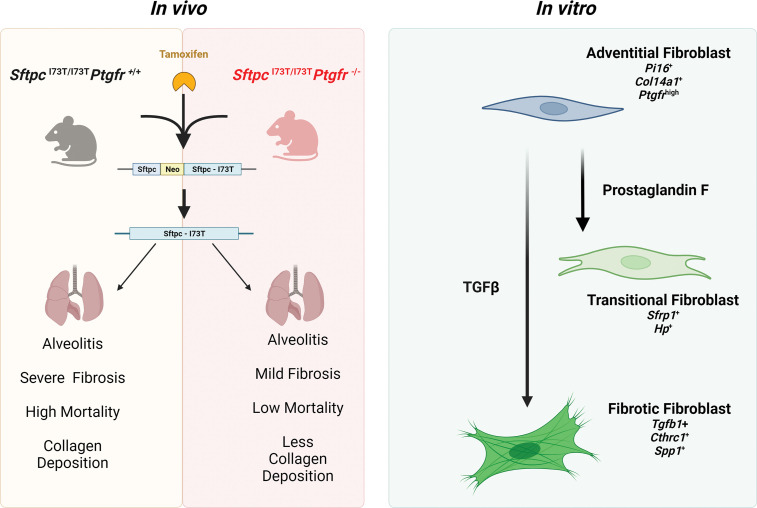

## Introduction

Idiopathic pulmonary fibrosis (IPF) is the most common subtype within a larger family of fibrosing parenchymal lung diseases in older adults (1, 2). The necessary IPF histology is the usual interstitial pneumonitis (UIP) pattern composed of temporally and spatially heterogeneous areas of fibroblast/myofibroblast accumulation coupled with extracellular matrix deposition, disruption of alveolar architecture, and subpleural honeycombing (3–5). The typical clinical experience resulting from unchecked fibroproliferation of patients with IPF is marked by progression of symptoms from cough and dyspnea to end-stage respiratory insufficiency resulting in lung transplantation or death within 3–5 years of diagnosis (6, 7). While 2 FDA-approved antifibrotic agents (pirfenidone; nintedanib) emerged in 2014 (8, 9), the collective experience in IPF drug discovery and development over the past 30 years has been an overwhelming series of late failures in clinical trials highlighting the continued unmet need as well as an incomplete understanding of its complex underlying pathobiology (10, 11).

Although knowledge gaps in IPF pathogenesis persist, the past 2 decades have seen progress with a paradigm shift that offers hope for new IPF discovery. Consensus has pushed the molecular origins of IPF toward a pivotal role for alveolar epithelial cells as a primary upstream driver of aberrant injury and repair (12, 13). Specifically, the postulated contributions of alveolar type 2 (AT2) cell dysfunction, along with disrupted alveolar niche cellular crosstalk, to the development of a fibrotic lung phenotype has gained momentum wherein microfoci of repeated cycles of AT2 injury produce a dysfunctional repair process reminiscent of other fibrotic diseases observed in skin, kidney, and liver (14, 15). Throughout this evolution, there has been no doubt about the role of fibroblasts as the pathological extracellular matrix–producing populations. With the emergence of single-cell RNA-Seq (scRNA-Seq), a complex heterogeneity in the fibroblast subpopulations that make up the IPF lung has been uncovered, providing identification and classification of multiple *Col1a1^+^* fibroblast subsets capable of participating in fibrogenic remodeling (16–23). Moreover, beyond the diseased environment, lung fibroblast heterogeneity is highly pertinent to homeostatic and developmental lung biology, considering that the role for fibroblast subpopulations in direct support of the epithelium is clear (24–27). Thus, critical concerns for IPF to be addressed include how a supportive mesenchyme enters a pathological state and what signaling pathways may promote this disruption.

Among the postulated mediators of crosstalk between these cellular components in the fibrotic niche, TGF-β along with PDGF, CTGF, and FGFs have received major attention (11). While antagonizing TGF-β has shown some therapeutic promise, off-target effects associated with blockade of its activation or signal transduction remain barriers to translation to the clinic (28). Similarly, in both preclinical models and clinical practice, neither pirfenidone nor nintedanib completely attenuate fibrotic endpoints or loss of lung function, suggesting additional pathways to fibrogenesis are contributors (8, 9, 29, 30).

Prostaglandins (PGs), secretory lipid mediators generated from arachidonic acid (AA), are multifaceted molecules that play critical roles in physiological balance, inflammation, and fibrosis (31). One PG in particular, PGF_2α_, signaling through its cognate F-prostanoid receptor (FPr), has emerged as a potential facilitator of lung fibrogenesis. In a preclinical genetic model, the fibrotic response to exogenous bleomycin-induced injury was attenuated in *Ptgfr*-null (FPr-null) mice (32). Furthermore, PGF_2α_ metabolites are elevated in plasma of patients with IPF (32, 33). While PGF_2α_ has also been shown in vitro to prompt fibroblast proliferation and collagen production in a TGF-β1–independent fashion, neither the effect size, specific PGF_2α_-dependent fibroblast subpopulations, nor the integration of this signaling axis into other preclinical PF models or human IPF have been defined.

Given the realities above, the complexities of IPF as a polycellular disease, and the many issues imparted by the use of exogenous injury models, we set out to define further the role of the PGF_2α_ axis in fibrotic lung remodeling by employing our murine model of spontaneous epithelial-driven lung fibrosis (I^ER^-*Sftpc^I73T^*) shown to recapitulate both pathological and clinical features of the human IPF/UIP (34). Using both a genetic and pharmacologic approach, we defined a role for PGF_2α_/FPr signaling in lung fibrosis, demonstrating an effect size for PGF_2α_ antagonism that is noninferior to that observed with nintedanib. Furthermore, scRNA-Seq analysis of the I^ER^-*Sftpc^I73T^* model detected the emergence of a *Col1a1* fibroblast subpopulation, which is dependent upon PGF_2α_/FPr signaling. Taken together, the results presented here implicate a role for PGF_2α_ as a mediator of downstream events in the pathogenesis of IPF through modulation of fibrogenic programs in mesenchymal subpopulations.

## Results

### Disruption of PGF_2α_ signaling reduces morbidity, mortality, and fibrotic endpoints in Sftpc^I73T^ mice.

**Figure 1:**
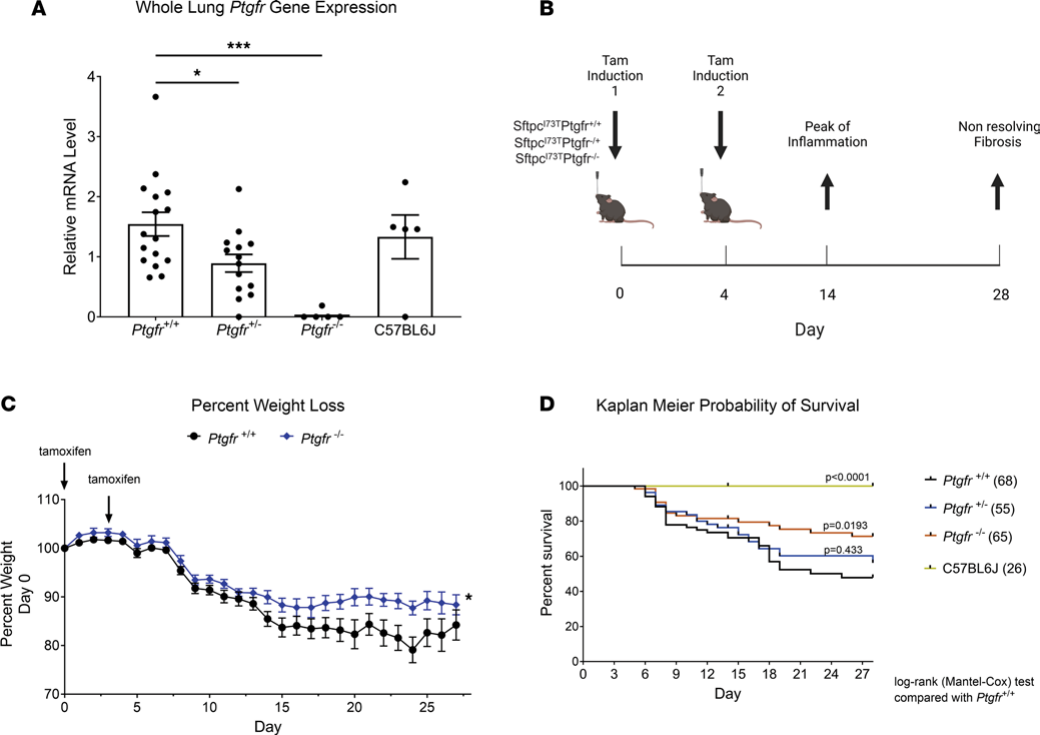
Deletion of *Ptgfr* reduces morbidity and mortality in I^ER^-*Sftpc^I73T^* mice. (**A**) *Ptgfr* mRNA content of whole lung mRNA isolated from generated lines of I^ER^*-Sftpc^I73T^/Ptgfr* mice deficient in 0, 1, 2 *Ptgfr* alleles assayed by qPCR. Ordinary 1-way ANOVA testing was performed, with statistical significance between groups denoted by *P < 0.05 and **P < 0.005. (**B**) Schematic of single, split i.p. or split OG dosing strategy employed for TAM induction of I^ER^*-Sftpc^I73T^*/*Ptgfr^–/–^* and I^ER^*-Sftpc^I73T^*/FPr^+/+^ cohorts. (**C**) Representative weight loss curve from a single cohort containing I^ER^*-Sftpc^I73T^*/*Ptgfr^–/–^* (*n* = 20) and I^ER^*-Sftpc^I73T^*/*Ptgfr^++–^*(*n* = 13) controls; mixed-effects modeling was performed with time x genotype. *P < 0.05. (**D**) Aggregate Kaplan-Meier curve for I^ER^*-Sftpc^I73T^*/*Ptgfr^–^* mice from 3 cohorts separately induced with either single i.p. or split i.p. doses of TAM in corn oil, with total numbers of each *Ptgfr* genotype shown. Negative controls consisted of Sftpc^WT^ C57BL/6 mice given TAM or uninduced I^ER^*-Sftpc^I73T^*/*Ptgfr^–/–^* animals. *P* values versus I^ER^*-Sftpc^I73T^*/*Ptgfr^–/–^* obtained by log-rank testing are shown.

As reported, the I^ER^-*Sftpc^I73T^*–knockin mouse is a model of spontaneous lung fibrosis generated by “inducible” expression of the disease-associated mutant SP-C^I73T^ protein from a single dose of i.p. administered tamoxifen (TAM) (34). To assess the role of PGF_2α_ signaling in pulmonary fibrosis, we first employed a genetic approach by crossing *Sftpc^I73T^* mice to a *Ptgfr*-deficient line (Supplemental Figure 1; supplemental material available online with this article; https://doi.org/10.1172/jci.insight.172977DS1) with resultant genotypes lung *Ptgfr* mRNA levels reflecting the loss of 1 or 2 alleles (Figure 1A) and without changes in overall urinary prostanoid levels (Supplemental Figure 1B). Challenge of the resultant genotypes with induction of *Sftpc*^I73T^ was performed using TAM dissolved in corn oil delivered either via oral gavage (OG) (Day 0 [D0], D4) or by i.p. injection (D0, D3) (Figure 1B). Regardless of route of administration (Supplemental Figure 2A), each induction modality resulted in similar degrees of weight loss at 14 days (Supplemental Figure 2B) as well as comparable levels of mutant *Sftpc^I73T^* gene expression (Supplemental Figure 2C) and bronchoalveolar lavage fluid (BALF) cell counts (Supplemental Figure 2D) at 28 days that were equivalent to single i.p. dosing of I^ER^*-Sftpc^I73T^/Ptgfr^+/+^* controls. However, TAM induction of I^ER^*-Sftpc^I73T^/Ptgfr^–/–^* resulted in decreased weight loss compared with I^ER^*-Sftpc^I73T^/Ptgfr^+/+^* controls (Figure 1C), and Kaplan-Meier analysis encompassing multiple cohorts demonstrated a *Ptgfr* allelic gene dose-dependent effect on survival (Figure 1D).

**Figure 2:**
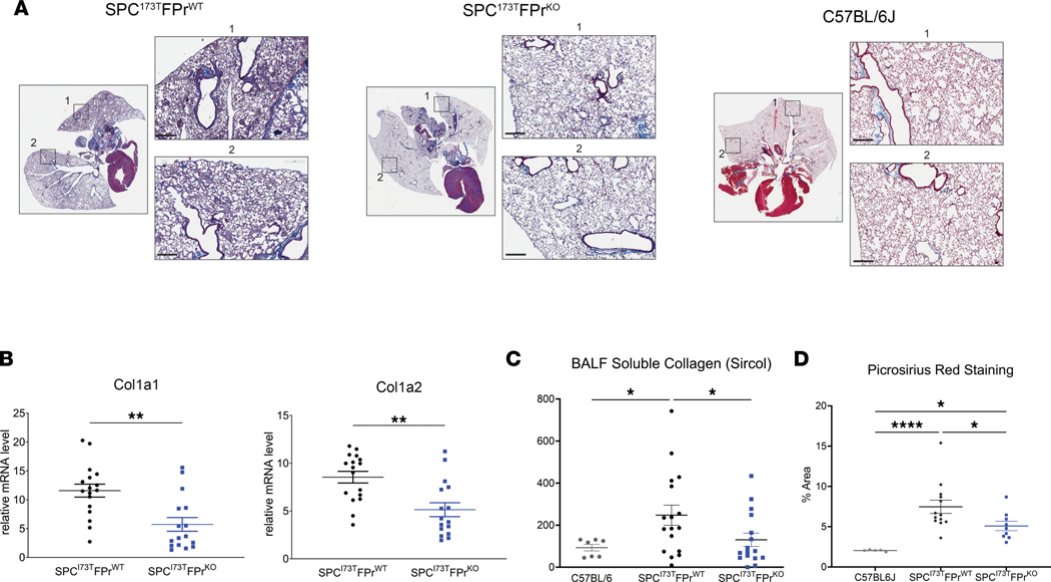
*Ptgfr* deficiency mitigates collagen expression and deposition after induction of *Sftpc^I73T^*. (**A**) Representative histology from I^ER^*-Sftpc^I73T^/Ptgfr^+/+^* and I^ER^*-Sftpc^I73T^/Ptgfr^–/–^* mice 28 days after TAM and development of fibrosis. Images are derived from Masson’s trichrome–stained sections. Scale bars: 300 μM. (**B**) Relative fold mRNA levels between I^ER^*-Sftpc^I73T^/Ptgfr^+/+^* and I^ER^*-Sftpc^I73T^/Ptgfr^–/–^* measured via qPCR demonstrates decreased *Col1a1* and *Col1a2* in I^ER^*-Sftpc^I73T^/Ptgfr^–/–^* mice 28 days after TAM induction. Statistical significance testing was performed using 2 tailed Welch’s *t* test. **P < 0.005. (**C**) Quantification of soluble collagen in BALF from mice during fibrotic remodeling reveals a lower concentration in I^ER^*-Sftpc^I73T^/Ptgfr^–/–^* mice. (**D**) PSR staining for collagen fibrils indicates mitigation of collagen deposition in I^ER^*-Sftpc^I73T^/Ptgfr^–/–^* mice. Quantification was performed using ImageJ; data represent percentage of total section area. All quantified data in this figure are derived from I^ER^*-Sftpc^I73T^/Ptgfr^+/+^* (*n* = 17) and I^ER^*-Sftpc^I73T^/Ptgfr^–/–^* (*n* = 16). In **C** and **D**, ordinary 1-way ANOVA testing was performed. *P < 0.05, ****P < 0.00005.

I^ER^*-Sftpc^I73T^/Ptgfr*–null animals were significantly protected from *Sftpc*^I73T^-induced pulmonary fibrosis. Lung sections prepared from D28 animals showed marked changes in Masson’s trichrome collagen staining (Figure 2A) that were accompanied by quantitative reductions in collagen gene expression, soluble collagen, and fibrillar collagen staining of lung sections (Figure 2, B–D).

The effect size of these changes was like that observed using the FDA-approved antifibrotic nintedanib in the same I^ER^-*Sftpc^I73T^* preclinical model (Supplemental Figure 3). Following oral TAM induction, mice were randomized on D12 to receive either nintedanib or vehicle in a “treatment intervention” protocol (Supplemental Figure 3A). By D28, nintedanib treatment resulted in significant reductions in BALF total protein, BALF soluble collagen, and lung fibrillar collagen as assessed by picrosirius red (PSR) staining (Supplemental Figure 3, D–F) accompanied by improvement in lung histology (Supplemental Figure 3G). The magnitude of nintedanib-mediated changes in these endpoints was consistent with prior published data in bleomycin-injured mice (30), as were the observed minor improvements in weight loss, BALF cell counts, and restrictive lung physiology (Supplemental Figure 3, B, C, and G).

### Nintedanib is not additive to FPr signaling in modulating fibrotic endpoints.

**Figure 3:**
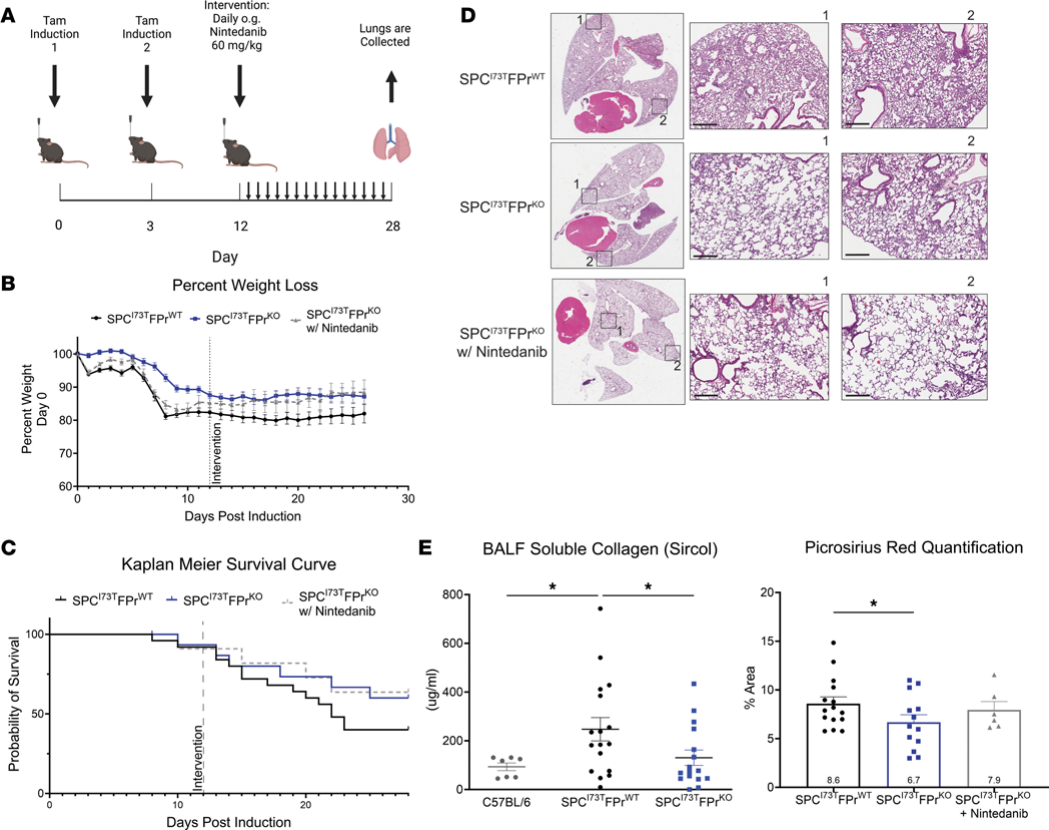
Nintedanib intervention is not additive to *Ptgfr* deficiency in I^ER^*-Sftpc^I73T^* mice. (**A**) Daily nintedanib intervention (60 mg/kg) was initiated at D12 after TAM induction. Following 16 days of intervention, surviving mice were euthanized and processed to evaluate fibrotic endpoints. (**B**) Weight loss as a percent of starting weight was tracked throughout the study; nintedanib intervention in I^ER^*-Sftpc^I73T^/Ptgfr^–/–^* mice did not reduce mean weight loss. (**C**) Kaplan-Meier survival analysis by log-rank testing demonstrates a nonsignificant improved probability of survival in I^ER^*-Sftpc^I73T^/Ptgfr^–/–^* that was not improved through nintedanib intervention. (**D**) Representative histology from I^ER^*-Sftpc^I73T^/Ptgfr^+/+^*, I^ER^*-Sftpc^I73T^/Ptgfr^–/–^*, and nintedanib-treated I^ER^*-Sftpc^I73T^/Ptgfr^–/–^* mice 28 days after TAM and development of fibrosis. Images are derived from H&E-stained sections. Scale bars: 300 μM. (**E**) Soluble collagen in BALF as measured by Sircol assay and fibrillar collagen in histological sections measured by PSR staining demonstrated a significant decrease in I^ER^*-Sftpc^I73T^/Ptgfr^–/–^* mice; again, nintedanib treatment did not improve these outcomes. Quantification of PSR was performed using ImageJ, and data represent percentage of total section area. Survival and weight loss data are derived from I^ER^*-Sftpc^I73T^/Ptgfr^+/+^* (*n* = 26), I^ER^*-Sftpc^I73T^/Ptgfr^–/–^* without nintedanib (*n* = 12), and I^ER^*-Sftpc^I73T^/Ptgfr^–/–^* with nintedanib (*n* = 12). Soluble collagen and PSR analysis included I^ER^*-Sftpc^I73T^/Ptgfr^+/+^* (*n* = 14), I^ER^*-Sftpc^I73T^/Ptgfr^–/–^* without nintedanib (*n* = 11), and I^ER^*-Sftpc^I73T^/Ptgfr^–/–^* with nintedanib (*n* = 7). Ordinary 1-way ANOVA testing was performed. *P < 0.05.

We next tested the combined efficacy of nintedanib and FPr signaling inhibition in our genetic model. Using a 3-armed protocol, cohorts of I^ER^*-Sftpc^I73T^/Ptgfr^+/+^* and I^ER^*-Sftpc^I73T^/Ptgfr^–/–^* animals were induced with oral TAM and allowed to progress through an inflammatory stage. As the mice transitioned into fibrogenesis (D12), the I^ER^*-Sftpc^I73T^/Ptgfr^–/–^* cohort was randomized to receive either nintedanib or vehicle (Figure 3A). As expected, I^ER^*-Sftpc^I73T^/Ptgfr^–/–^* mice were protected from weight loss and late mortality (Figure 3, B and C) compared with I^ER^*-Sftpc^I73T^* *Ptgfr^+/+^* animals; however, addition of nintedanib provided no added improvement in morbidity, mortality, or any fibrotic endpoint including BALF soluble collagen (Figure 3E [left]), fibrillar collagen deposition (Figure 3E [right]), or lung histology (Figure 3D and Supplemental Figure 4C).

To corroborate these data sets, the protective effect of PGF_2α_ signaling on lung fibrogenesis was also assessed pharmacologically using FPr antagonist tool compounds in a model of bleomycin-induced lung fibrosis (Supplemental Figure 4B). Both OBE022 and BAY6672 have been shown to exhibit potent and selective blockade of FPr-mediated signaling (35, 36). Using an intervention protocol, mice challenged with intratracheal bleomycin were randomized to receive drug or vehicle beginning on D6 and animals were euthanized on D22 (Supplemental Figure 4A). In a study focused on histological endpoints, BAY6672 delivered twice daily (30 and 100 mpk) produced reductions in collagen staining, Ashcroft scoring, and α-SMA staining at magnitudes like nintedanib (Supplemental Figure 4B). In a separate experiment using OBE022, significant changes in body weight were not observed at each of 2 doses (100 and 300 mpk). However, reductions in BALF soluble collagen and cell counts similar in magnitude to nintedanib were observed, while reductions in histological fibrosis scores were inferior to nintedanib (Supplemental Figure 4C).

Taken together, these data are consistent with the hypothesis that FPr blockade produces an antifibrotic effect size similar to but not additive to nintedanib.

### Ptgfr deficiency had no effect on early lung inflammation induced by SP-C^I73T^.

**Figure 4:**
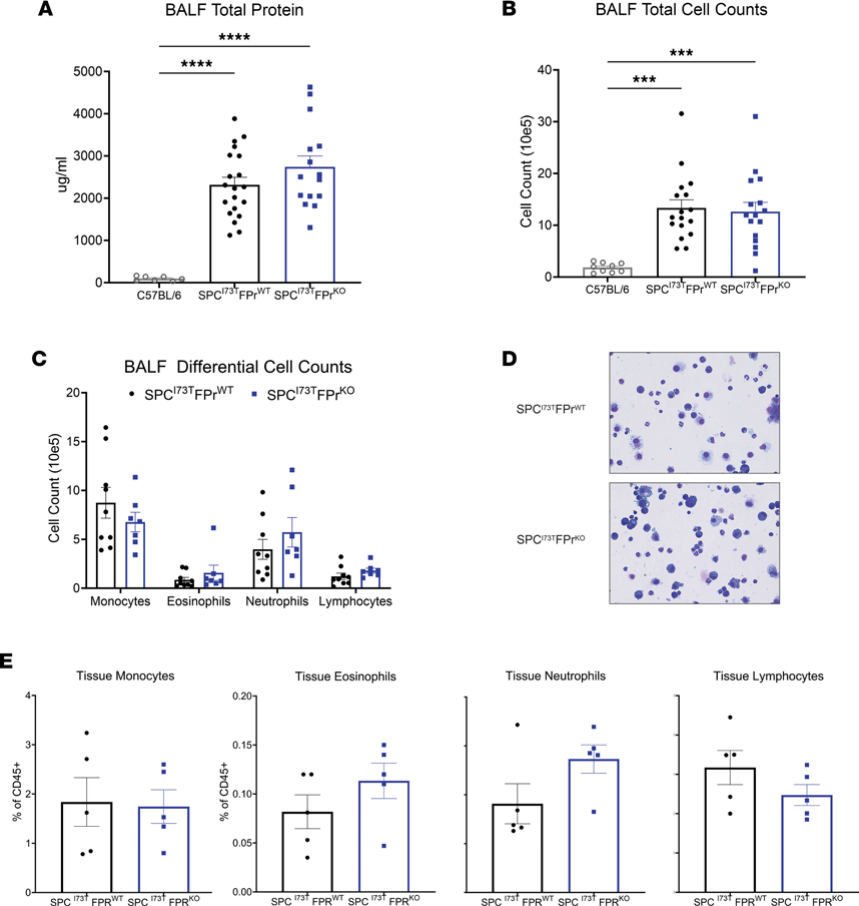
*Ptgfr* deficiency has no effect on early lung injury and inflammation in I^ER^-*Sftpc^I73T^*. (**A** and **B**) Quantification of total protein and total cell counts in BALF did not result in a significant difference between I^ER^*-Sftpc^I73T^/Ptgfr^+/+^* (*n* = 17) and I^ER^*-Sftpc^I73T^/Ptgfr^–/–^* (*n* = 16) mice 14 days after TAM induction. Ordinary 1-way ANOVA testing was performed. ***P < 0.005, ****P < 0.0005. (**C**) BALF cell differential determined by quantification of modified Giemsa-stained cytospins yielded no significant difference between I^ER^*-Sftpc^I73T^/Ptgfr^+/+^* (*n* = 9) and I^ER^*-Sftpc^I73T^/Ptgfr^–/–^ (n* = 7*)* mice 14 days after TAM induction. (**D**) Representative Giemsa-stained images from I^ER^*-Sftpc^I73T^/Ptgfr^+/+^* and I^ER^*-Sftpc^I73T^/Ptgfr^–/–^* mice 14 days after TAM induction. Total original magnification, ×100. (**E**) Flow cytometry quantification of whole-lung single-cell suspensions confirms that there is no differential immune cell infiltration between I^ER^*-Sftpc^I73T^/Ptgfr^+/+^* (*n* = 5) and I^ER^*-Sftpc^I73T^/Ptgfr^–/–^*(*n* = 5) mice 14 days after TAM induction. Ordinary 1-way ANOVA testing was performed for this analysis.

To exclude the possibility that the observed antifibrotic effect of FPr signaling blockade were related to upstream effects on lung inflammation/injury, we surveyed the I^ER^*-Sftpc^I73T^/Ptgfr* model 14 days after TAM (the published peak of early inflammatory phase). As shown in Figure 4, we found that the absence of *Ptgfr* signaling produced no reduction in either BALF protein or total cell counts (Figure 4, A and B). Differential counting of BALF cytospins revealed no alterations in the distribution of monocytes, eosinophils, neutrophils, or lymphocytes (Figure 4, C and D). We also assessed immune populations in the lung parenchyma by flow cytometry analysis using a previously published protocol illustrated in Supplemental Figure 5, A and C (34, 37, 38). When compared with *Ptgfr^+/+^* controls, at D14, the induced I^ER^*-Sftpc^I73T^/Ptgfr^–/–^* cohort had similar levels of both myeloid and lymphocyte lineages (Figure 4E and Supplemental Figure 5B).

### scRNA-Seq analysis identifies increased Ptgfr expression in adventitial fibroblasts.

**Figure 5:**
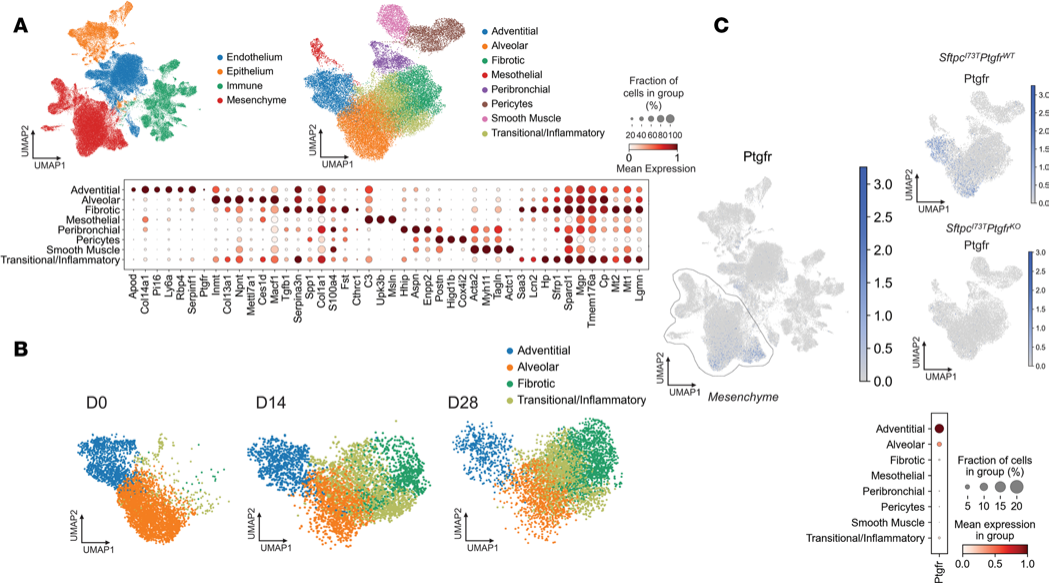
*Ptgfr* expression is limited to adventitial and alveolar fibroblasts. (**A**) UMAP clustering 94,258 cells identifies 4 primary cell compartments in I^ER^*-Sftpc^I73T^/Ptgfr^+/+^*, I^ER^*-Sftpc^I73T^/Ptgfr^–/–^*, and uninduced controls. Subclustering of the mesenchymal compartment identifies 8 mesenchymal clusters defined by marker genes depicted as a gradient dot plot. (**B**) UMAP projections of *Pdgfra*^+^ mesenchymal populations across time identifies 2 injury-specific clusters (fibrotic and transitional/inflammatory). (**C**) UMAP projection of all cells identifies the restriction of *Ptgfr* expression to the mesenchymal compartment and lack of *Ptgfr* expression in I^ER^*-Sftpc^I73T^/Ptgfr^–/–^* mice. Gradient dot plot of *Ptgfr* expression within the mesenchyme demonstrates increased expression and increased percent expression of *Ptgfr* in adventitial fibroblasts as compared with alveolar fibroblasts.

A prior study has demonstrated that PGF_2α_ treatment modifies fibroblast phenotypes in vitro (32). Given the immense heterogeneity within lung stromal cells at homeostasis and the dynamic changes in both surface markers and transcriptomic profiles that occur during fibrogenesis in other in vivo model systems (e.g., bleomycin) and human IPF (19, 21, 23, 24, 26, 27, 39, 40), we next performed scRNA-Seq on digested lung samples to assess transcriptional differences in specific mesenchymal subsets of the I^ER^*-Sftpc^I73T^* model in the presence and absence of PGF_2α_*-Ptgfr* signaling (Figure 5). We induced cohorts of I^ER^*-Sftpc^I73T^/Ptgfr^+/+^* and I^ER^*-Sftpc^I73T^/Ptgfr^–/–^* animals with oral TAM and harvested the lungs at 14 and 28 days (*n* = 2 per genotype per time point). Controls (*n* = 4) consisted of 1 mouse each of uninduced I^ER^*-Sftpc^I73T^/Ptgfr^+/+^*, I^ER^*-Sftpc^I73T^/Ptgfr^–/–^*, *Sftpc^WT^/Ptgfr^+/+^*, and *Sftpc^WT^/Ptgfr^–/–^* genotypes. To balance stromal and effector cell numbers, resultant single-cell suspensions were initially depleted using α-CD45 magnetic beads, and captured CD45 cells then “spiked back” to achieve approximately 20% of the total cell numbers.

We profiled 35,002 cells from I^ER^*-Sftpc^I73T^/Ptgfr^+/+^*, 32,594 cells from I^ER^*-Sftpc^I73T^/Ptgfr^–/–^*, and 26,662 cells from pooled control animals and identified all major populations including endothelial, epithelial, mesenchymal, and effector cell populations (Figure 5A and Supplemental Figure 6). Removing non–mesenchymal cell (non-MC) populations defined using marker genes from multiple published studies and publicly available gene sets (Supplemental Figure 7), we observed 8 well-segregated clusters of MC (Supplemental Figure 8), and each expressed a unique profile of marker genes (Figure 5A). These included alveolar fibroblasts or lipofibroblasts (*Npnt*, *Inmt*, *Ces1d*), adventitial fibroblast (*Col14a*, *Pi16*, *Apod*), fibrotic fibroblast (*Col1a1^hi^*,*Tgfb1*, *Spp1*, *Cthrc1*), and a “transitional/inflammatory” population (*Sfrp1*, *Lcn2*, *Saa3*, *Hp*) recently defined by several groups (19–23). When stratified by time from induction of *Sftpc^I73T^* model, most MC at D0 (uninduced or control) were in the alveolar and adventitial clusters. TAM induction resulted in time-dependent increases in transitional/inflammatory and fibrotic populations commensurate with a loss of alveolar and adventitial cells (Figure 5B and Supplemental Table 3). Importantly, among these 8 MC populations, *Ptgfr* expression predominantly localized in the adventitial fibroblast population, with a lesser degree in the alveolar cluster of *Ptgfr* genotypes (Figure 5C). We also failed to detect significant *Ptgfr* expression in endothelial, epithelial, or immune cell clusters (Figure 5C)

### Reprogramming of adventitial fibroblasts to the transition/inflammatory state is Ptgfr dependent.

**Figure 6:**
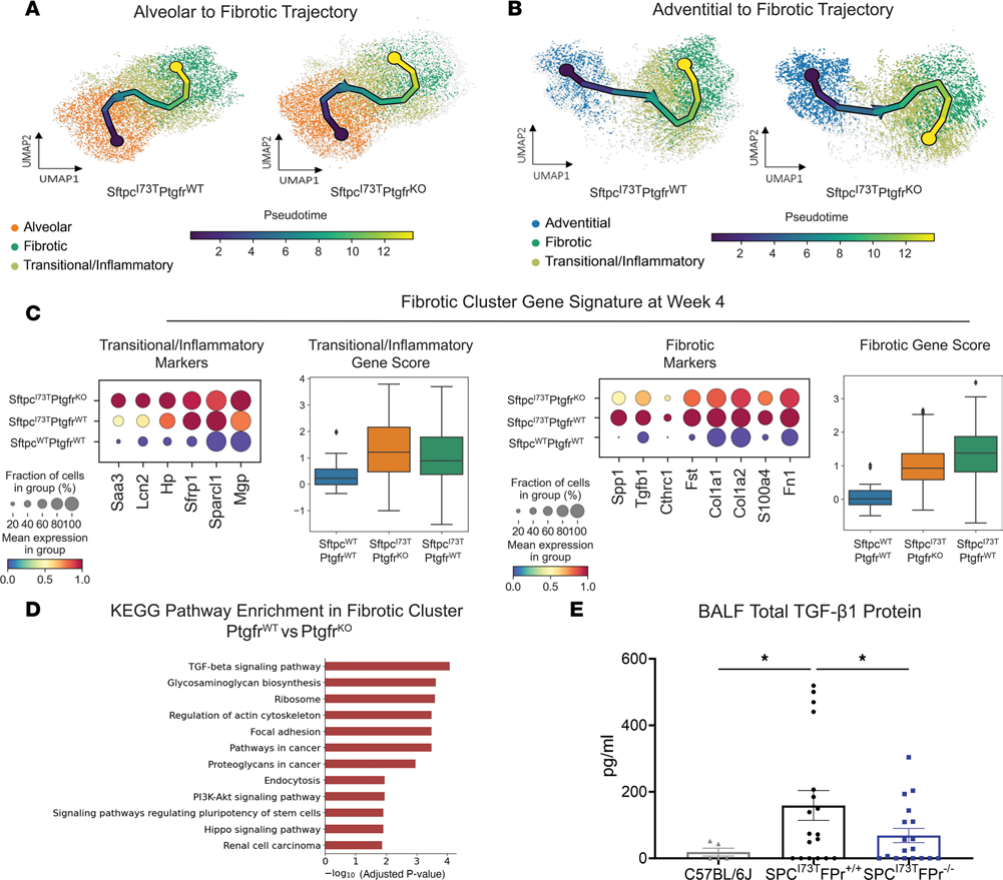
*Ptgfr* deficiency alters fibroblast lineage trajectory through fibrotic remodeling. (**A**) UMAP analysis of reclustered alveolar, transitional/inflammatory, and fibrotic fibroblasts with superimposed vector from pseudotime trajectory analysis reveals no *Ptgfr*-dependent effect on terminal node. (**B**) UMAP analysis of reclustered adventitial, transitional/inflammatory, and fibrotic fibroblasts with superimposed vector from pseudotime trajectory analysis demonstrates a *Ptgfr*-dependent effect on terminal node. In I^ER^*-Sftpc^I73T^/Ptgfr^–/–^*, the terminal node is found in the transitional/inflammatory cluster, while *Sftpc^I73T^/Ptgfr^+/+^* samples have a vector terminating in the fibrotic cluster. (**C**) Comparative analysis of gene expression within the fibrotic cluster of I^ER^*-Sftpc^I73T^/Ptgfr^+/+^* and I^ER^*-Sftpc^I73T^/Ptgfr^–/–^* mice is presented by gradient gene expression dot plots. Genes seen in dot plots were combined to generate a score and plotted in a box plot. Marker genes associated with the transitional/inflammatory cluster are comparatively elevated in I^ER^*-Sftpc^I73T^/Ptgfr^–/–^* mice, while fibrotic marker genes are elevated in the I^ER^*-Sftpc^I73T^/Ptgfr^+/+^* mice. All comparisons achieved statistical significance. (**D**) KEGG pathway enrichment analysis comparing the fibrotic clusters identifies multiple pathways associated with cytoskeletal rearrangement, mesenchymal activation, and TGF-β signaling that are upregulated in I^ER^*-Sftpc^I73T^/Ptgfr^+/+^* mice. (**E**) Measurement of BALF TGF-β1 via ELISA demonstrates a significant decrease in I^ER^*-Sftpc^I73T^/Ptgfr^–/–^* mice (*n* = 18) as compared with I^ER^*-Sftpc^I73T^/Ptgfr^+/+^* mice (*n* = 18). Ordinary 1-way ANOVA testing was performed. *P < 0.05.

The restriction of *Ptgfr* expression to the mesenchymal compartment, specifically adventitial and alveolar fibroblasts (Figure 5C), facilitated an assessment of the role of PGF_2α_ signaling in lung fibroblast populations during the evolution of pulmonary fibrosis. To identify the potential progenitors of the fibrotic population ,we performed pseudotime analysis using scFates (Figure 6, A and B). Vectors originating from alveolar fibroblasts suggest entry into the transitional population prior to terminating in the fibrotic cluster and were unaffected by the loss of *Ptgfr* (Figure 6A). In contrast, while I^ER^*-Sftpc^I73T^/Ptgfr^+/+^* adventitial fibroblasts were shown to be candidate progenitors for terminal fibrotic fibroblasts, the *Ptgfr*^–/–^ adventitial population was markedly disrupted with the terminal node of this vector predicted within the transitional cluster suggesting a failure to progress (Figure 6B). Supporting the pseudotime analysis, gene expression profiles of the resulting fibrotic gene clusters from I^ER^*-Sftpc^I73T^/Ptgfr^+/+^*, I^ER^*-Sftpc^I73T^/Ptgfr^–/–^*, and *Sftpc^WT^*/Ptgfr^+/+^ populations at D28 demonstrated that I^ER^*-Sftpc^I73T^/Ptgfr^–/–^* fibrotic clusters were relatively deficient in the fibrotic signature genes while retaining increased levels of transitional genes, suggesting that a maximal fibrogenic response required intact PGF_2α_ signaling (Figure 6C). Gene set enrichment analysis (GSEA; using KEGG) comparing the fibrotic clusters displayed a transcriptomic profile consistent with an attenuated fibrotic signature and decreased TGF-β signaling in FPr-KO mice (Figure 6D). Looking specifically at *Tgfb1* expression in the lung, mesenchymal-specific expression was decreased in *Ptgfr*^–/–^ mice (Supplemental Figure 9, C and D). Interestingly, all other major lung compartments (immune, epithelial, endothelial) in *Ptgfr*^–/–^ mice also present with reduced *Tgfb1* expression consistent with an important role for *Tgfb1* in the development of fibrosis in this model (Supplemental Figure 9, D and E). Finally, analysis of TGF-β1 in BALF identifies a *Ptgfr-*dependent decrease in TGF-β1 (Figure 6E), confirming the transcriptomic analysis and suggesting that terminal fibrotic fibroblasts are a primary source of TGF-β1 in the *Sftpc^I73T^* model.

**Figure 7:**
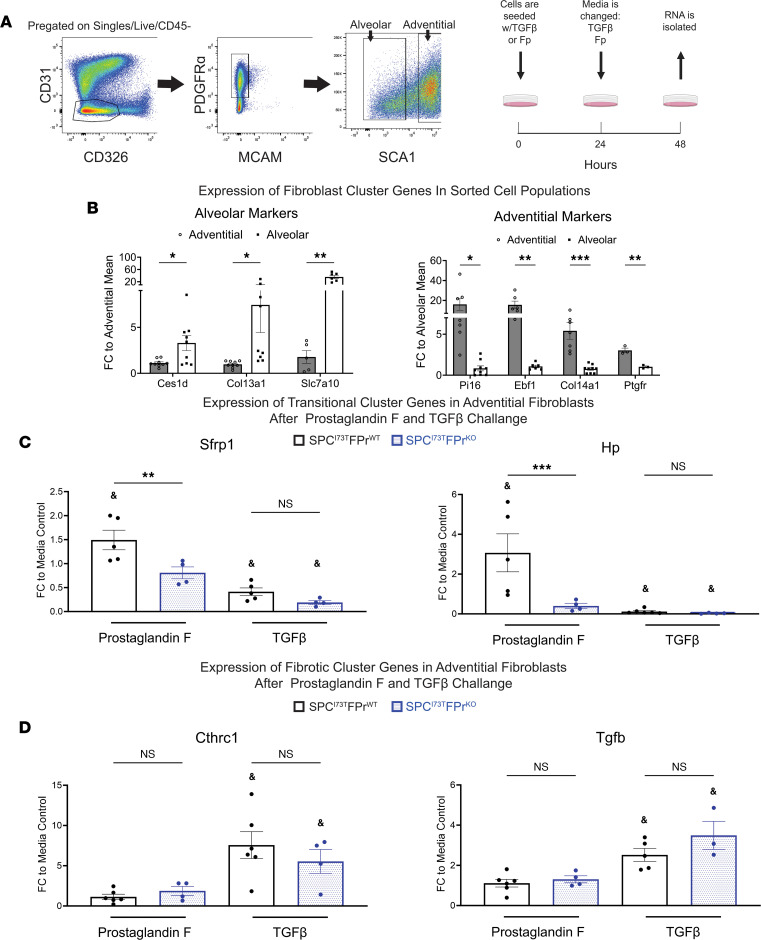
In vitro prostaglandin F2α (PGF_2α_) challenge promotes adventitial fibroblast entry into the transitional/inflammatory state. (**A**) Sorting strategy for the isolation of adventitial and alveolar fibroblast used in I^ER^*-Sftpc^I73T^/Ptgfr^+/+^* and I^ER^*-Sftpc^I73T^/Ptgfr^–/–^* prior to induction by TAM. Initial gating was performed on the CD45^–^CD31^–^CD326^–^Mcam^–^Pdgfra^+^ population. Adventitial fibroblasts are Sca1^+^, and the Sca1^–^ population is made up of the alveolar fibroblasts. After sorting, cells were seeded for 48 hours on tissue culture plastic with either 10 ng/mL TGF-β1, 500 nM PGF_2α_, or media control. (**B**) Gene expression analysis via qPCR in untreated adventitial and alveolar fibroblasts quantifying the expression of population-specific marker genes confirms the identity of target fibroblasts. (**C**) Quantification of transitional cluster maker genes after 48-hour challenge demonstrates the potential for FPr to induce the transitional state in adventitial fibroblasts. This is not observed in adventitial fibroblasts lacking the FPr. (**D**) Quantification of fibrotic cluster marker genes after 48-hour challenge confirms that TGF-β promotes entry adventitial fibroblasts into the fibrotic state independent of FPr status. Ordinary 1-way ANOVA testing was performed. *P < 0.05, **P < 0.005, ***P < 0.0005. Statistical significance between treatment and media control denoted by ^&^*P* < 0.05.

To validate the computational analysis, we next assessed the ability of PGF_2α_ signaling to promote adventitial entry into the transitional state in vitro (Figure 7). Employing cell surface markers identified in prior published studies (19–21), we first isolated adventitial and alveolar populations from bulk mesenchyme prepared from *Sftpc^WT^/Ptgfr^+/+^* and *Sftpc^WT^/Ptgfr^–/–^* mice using FACS (Figure 7A). When analyzed by quantitative PCR (qPCR), the resultant mesenchymal populations (alveolar and adventitial) were each enriched in population-specific marker genes (Figure 7B). This analysis also confirmed the sustained increased expression of *Ptgfr* in the adventitial fibroblast after isolation. Consistent with the scRNA-Seq–derived bioinformatic predictions, after culture and challenge with PGF_2α_, adventitial but not alveolar fibroblasts acquired markers of the transition state (*Sfrp1^+^*/*Hp^+^*) that was FPr dependent (Figure 7C and Supplemental Figure 10). Notably, PGF_2α_ treatment of either population failed to stimulate markers associated with the fibrotic myofibroblast cluster, while both adventitial and alveolar fibroblast populations significantly increased pathologic fibrotic marker expression (*Cthrc1^+^/Col1a1^+^*) in response to TGF-β in a manner independent of FPr status. Interestingly, commensurate with these changes, TGF-β also downregulated markers associated with the transition/inflammatory state (Figure 7D). These data further support a role for PGF_2α_/FPr-mediated signaling in selectively modulating the transcriptomic trajectory of an important MC progenitor population capable of contributing to fibrogenesis.

## Discussion

Idiopathic pulmonary fibrosis remains a clinical challenge as an unmet need persists for well-tolerated and effective IPF therapeutics. While the fibroblast is recognized as a key lung effector cell population in IPF responsible for the synthesis and maintenance of extracellular matrix during aberrant injury repair, the emerging complexity of its biology, including the functional importance of recently identified diverse fibroblast subsets, has created new challenges for drug discovery. The bioactive eicosanoid, PGF_2α_, acting through its cognate receptor FPr has been implicated as a facilitator of fibrogenesis in IPF (32); however, a detailed understanding of the target mesenchymal populations influenced by PGF_2α_ in IPF is lacking. Thus, to assess the role of PGF_2α_/FPr signaling in IPF mechanistically, we utilized both genetic and pharmacologic approaches in 2 mouse models of IPF to affirm a contribution of FPr signaling to lung fibrogenesis. This established an effect size for this pathway equivalent, but not additive, to that observed with the clinical antifibrotic nintedanib. Then, using unbiased scRNA-Seq and in vitro validation, we localized *Ptgfr* expression predominantly within an adventitial fibroblast subpopulation that was capable of being selectively reprogrammed to a recently described “inflammatory/transitional” cell state in a PGF_2α_-dependent manner. Collectively, our findings support a role for PGF_2α_ signaling in IPF and mechanistically identify a target fibroblast subpopulation while also establishing a benchmark for disruption of this pathway in mitigating fibrotic lung disease.

For this study, the role of FPr signaling was first assessed in a clinically relevant model of spontaneous pulmonary fibrosis, the I^ER^-*Sftpc^I73T^* mouse, which we have shown to recapitulate many characteristic features of IPF, including histopathology, restrictive physiology, and biomarkers also found in human IPF. The translational relevance of this model was further supported by demonstration of the potential for nintedanib to partially rescue the fibrotic phenotype of the *Sftpc*^I73T^ mouse (Supplemental Figure 3), in line with both other preclinical models and the observed clinical experience (8, 9, 30). Leveraging this model in combination with concomitant genetic ablation of FPr (Supplemental Figure 1), we found that disruption of PGF_2α_ signaling conferred a survival advantage and attenuated the fibrotic burden similar in magnitude to that of nintedanib alone (Figures 1 and 2, and Supplemental Figure 3) here or in the bleomycin-challenged FPr-KO mouse (32). We then extended these findings using 2 pharmacologic inhibitors of FPr signaling administered in an intervention protocol in the bleomycin mouse model, which permitted the temporal segregation of early injury/ inflammation from late fibrosis (Supplemental Figure 4). OBE022, an FPr antagonist in clinical development as a tocolytic (35, 41), and BAY6672, a quinolone-based FPr antagonist shown to attenuate silica-induced lung fibrosis in mice (36), each blocked fibrotic endpoints with effect sizes similar to nintedanib alone. We further note that, while the mean effect of all compounds was similar, the range of responses within the groups of mice was variable and compound specific. This can be attributed to differences in pharmacology (pharmacokinetics/pharmacodynamics) and tolerability differences between these drugs. Further improvements to chemistry or delivery vehicles would likely decrease individual response variability. Importantly, a combination strategy of nintedanib with either genetic or pharmacological targeting of FPr signaling was not additive or synergistic for any fibrotic endpoint (Figure 3 and Supplemental Figure 4). While this was surprising given that the known triple receptor kinase targets of nintedanib are distinct from PGF_2α_/FPr signaling, our findings do not exclude that these 2 pathways can intersect at the same profibrotic cell population. Given current clinical trial design for potential IPF therapeutics, development of new first-in-class antifibrotics may not be as straightforward as simply targeting molecular pathways distinct from existing clinical therapies.

Despite the plethora of established and emerging targets for IPF, nearly all in vitro and in vivo approaches continue to measure efficacy based on the inhibition of the fibroproliferative state of the bulk lung mesenchyme (11, 42). With the rapid dissemination of single-cell transcriptomic technology, a refined understanding of the spatial localization and pathological behavior of fibroblasts throughout the aberrant remodeling in both human IPF and murine fibrosis platforms is emerging (19–23, 39). Specifically, from computational inference analysis, lineage tracing, and in vitro modeling, anywhere from 6 to 9 MC subtypes have now been described with pathological collagen-producing fibroblasts (*Cthrc1^+^* or myofibroblast). These may arise from several of these lineages within spatially distinct compartments of the lung, including the *Col13a1^+^Npnt^+^* alveolar (lipo) fibroblast of the proximal alveolar space as well as *Col14a1^hi^*/*Pi16^+^* MCs found in the lung adventitia, although the predominant cell of origin remains a point of contention (20, 43). Embedded in several of these studies is the observation that, under a variety of exogenous stimuli (cytokines, bleomycin injury), the trajectory to a “fibrotic” MC population may involve prior entry and exit from a profibrotic intermediate state conventionally designated as “transitional” or “inflammatory” (22, 23).

Using our *Sftpc^I73T^* fibrosis model, scRNA-Seq analysis supports and extends these observations by both affirming the identity of all previously identified MC populations (Figure 5) and establishing a fibrotic trajectory for both alveolar and adventitial fibroblast populations to pathological fibroblasts through an inflammatory/transition state in the absence of an exogenous injury (e.g., bleomycin). RNA velocity also revealed that only the trajectory of the adventitial population was disrupted by deletion of FPr (Figure 6A). Importantly, FPr deletion did not alter the early inflammatory response (Figure 3), supporting a mechanism of action at the level of the fibroblast.

The role for FPr signaling in fibroblast reprogramming events was further supported by the finding that, in the *Sftpc^I73T^* murine lung, *Ptgfr* expression occurred predominantly within the mesenchyme (Figure 5C), with the highest levels spatially restricted mainly to *Col14a1^hi^*/*Pi16^+^* adventitial fibroblasts. A minor component was found in the alveolar population (Figure 5C). Interestingly, *Col14a1^hi^*/*Pi16^+^* adventitial MCs, which may also represent the murine homologue of the previously identified Has1^+^ mesenchymal populations described in human IPF (17), were the sole fibroblast population that maintained *Ptgfr* expression throughout the fibrotic remodeling following induction of mutant *Sftpc^I73T^* (Supplemental Figure 9), raising the potential that PGF_2α_ signaling could modulate all or part of this trajectory in this population. This was confirmed using a reductionist approach isolating each of these populations and showing in vitro that it is the adventitial and not the alveolar population that demonstrates FPr dependence for entry into the transitional/inflammatory state (Figure 7). We note that, while significant expression of these genes was not observed in the alveolar population (Supplemental Figure 10), there was a small increase noted, which may be a result of the low level of alveolar fibroblast *Ptgfr* expression seen in both the single-cell data and later qPCR (Figure 5C and Figure 7B). In contrast to the mouse, FPr expression in humans is not solely limited to the mesenchyme. Both the Human IPF Cell Atlas (44) and publicly available IPF single-cell data sets (GSE135893) confirm expression of FPr in the mesenchyme; limited epithelial expression is also observed in humans. This limitation in mouse models suggests a second mechanism for FPr signaling in human disease but does not discount the implications for mesenchyme biology observed in our study.

**Figure 8:**
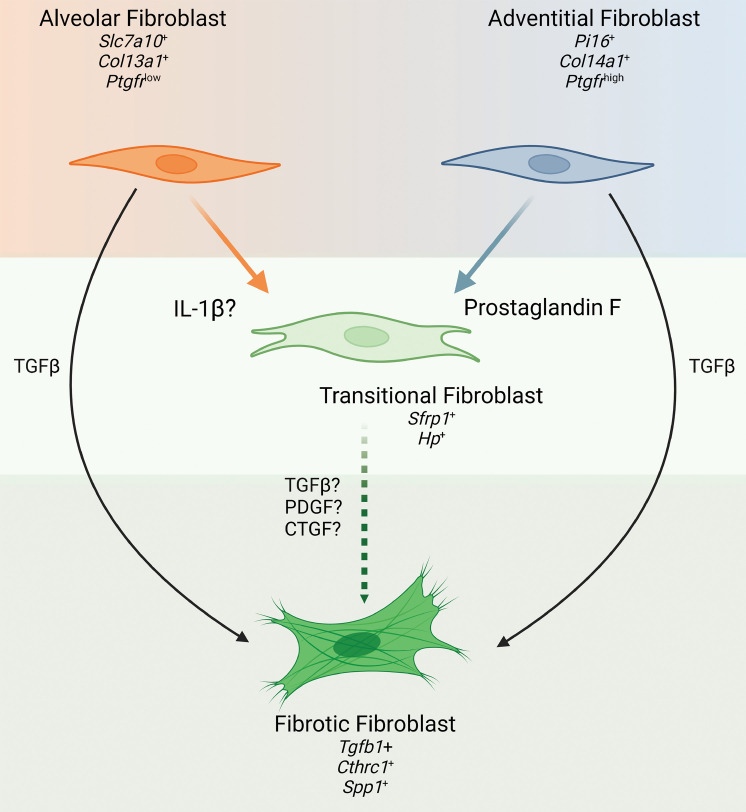
Role of PGF_2α_ as a driver of fibroblast heterogeneity in pulmonary fibrosis. Application of single-cell discovery and in vitro validation identifies PGF_2α_ as a driver of adventitial fibroblast activation into the transitional fibroblast state. Paired with work by Tsukui et al. demonstrating potential of alveolar (lipo) fibroblasts to enter the same transitional state through IL-1B stimulation (23), there is now evidence for differential drivers of transitional fibroblast differentiation within fibroblast population subsets. Concurrently, classical profibrotic TGF-β signaling drives entry of adventitial and alveolar fibroblasts as an “override” into a terminally activated fibroblast state expressing *Cthrc1*. Contextualizing these findings with previous work by Oga et al. (32) suggests that PGF_2α_-stimulated transitional fibroblast may be more sensitive to a second hit (e.g., TGF-β, PDGF, or CTGF), accelerating the expansion of the terminal fibrotic fibroblast population.

The granular analysis of fibroblast subpopulations and their trajectories viewed via single-cell analysis, combined with our in vitro observation that FPr signaling selectively induces the transitional/inflammatory state only in the adventitial fibroblast, likely indicates that a profibrotic (*Cthrc1^+^*/*Col1a1*^hi^) population can be derived from at least 2 precursor MC populations via a common intermediate state with entry governed by a variety of cues. As shown in Figure 8, for the adventitial fibroblast, PGF_2α_ represents at least 1 key driver of entry into a transitional/inflammatory state. We speculate that one or more of the signaling kinases inhibited by nintedanib would spatially overlap in the same adventitial population with resultant equivalency of effect size seen by their respective inhibition. This model does not preclude and, in fact, may support alternative fibroblasts, including the alveolar population, from generating profibrotic (*Cthrc1*) fibroblasts by arriving at the terminal profibrotic state through a separate signaling cascade (such as IL-1β) via the same or a parallel transitional/inflammatory state. It also provides a plausible explanation for why fibrogenesis is only partially attenuated, since there likely exists a large number of postinjury, proremodeling signals that each contribute to the development of the fibrotic phenotype through 1 or more of these precursor populations. Intervention in one or more of these signals may slow the development of a terminal fibrotic fibroblast population from a single compartment but does not comprehensively capture all pathways across multiple compartments that simultaneously influence human IPF.

We also note that, while PGF_2α_ promoted development of the transition state, it did not, in isolation, increase markers of the terminal profibrotic state. We also found that addition of TGF-β to either alveolar or adventitial fibroblasts in culture successfully drove each of these cells to a profibrotic state while decreasing expression of markers of the transitional/inflammatory state (Figure 7C and Supplemental Figure 10). The role of TGF-β as a potent profibrotic signal has been well documented and is a substantial area of interest for human clinical trials. In this study, we observed that TGF-β–expressing fibroblasts clustered into the terminal fibrotic population, and we have also shown previously that there is a significant amount of TGF-β in the fibrotic milieu arising from the immune compartment entering the profibrotic remodeling phase of injury resolution (34). Confirming these previous observations, we report increased *Tgfb1* expression in all major lung compartments (mesenchyme, immune, epithelial, and endothelial) and a decrease in this expression across all compartments in *Ptgfr*^–/–^ mice. This potent signal, arising from multiple sources, may represent a “system override” that increases the size of the fibrotic populations observed in our model by pushing cells rapidly through the transitional/intermediate state or present a second “hit” profibrotic signal to cells in the transitional state. Our in vitro data demonstrating the efficacy of TGF-β to induce the fibrotic state independent of an initial FPr signal in both adventitial and alveolar derived fibroblasts further reinforce the potential of this molecule to act independently of an upstream pathway driven by PGF_2α_. This finding expands on work by Oga et al. (32), where PGF_2α_ and TGF-β were each shown to increase collagen production and enhance proliferation in cultures of bulk isolated fibroblasts. We speculate that, in their model system that utilized heterogeneous bulk mesenchyme populations, PGF_2α_-mediated entry of the adventitial subpopulation into the transitional state was followed by a transitional state–dependent stimulation of a second fibroblast population that was not present in our purified populations. This would suggest an amplification of the injury response by transitional fibroblasts that may be driven by alternative profibrotic ligands such as connective tissue growth factor (CTGF) or platelet-derived growth factor (PDGF) and that may prompt wound repair. Confirmational studies of the trajectory and transcriptomic dynamics of the fibroblast populations will require the generation of cell population–specific reagents for lineage tracing as well as further genetic and/or pharmacologic interrogation of other pathways.

In conclusion, using the combined application of IPF genetic models and single-cell technology to address the fibroblast heterogeneity arising throughout the aberrant injury/repair process observed with pulmonary fibrosis, we have established a role for PGF_2α_ signaling in PF acting through a key mesenchymal population, the adventitial fibroblast. Our work here begins to elucidate the importance of the transitional profibrotic state that multiple fibroblast subpopulations enter and highlights the relevance to fibrotic remodeling when targeted through intervention strategies.

## Methods

### In vivo mouse models

#### Mouse model of TAM-induced Sftpc^I73T^ expression.

TAM-inducible *Sftpc*^I73T/I73T^ Rosa26ERT2FlpO^+/+^ (I^ER^-*Sftpc^I73T^*) mice expressing an NH_2_-terminal HA-tagged murine *Sftpc*^I73T^ mutant allele into the endogenous mouse *Sftpc* locus were previously generated as reported (34) and are detailed in Supplemental Methods. TAM treatment of adult I^ER^-*Sftpc^I73T^* mice was initiated at 12–14 weeks of age by either i.p. or OG as indicated. Both male and female animals were used for the studies.

#### Generation of FPr-deficient I^ER^-Sftpc^I73T^ mouse model.

Homozygous FPr-KO mice have been previously described (32) and were provided by Shuh Narumiya (Kyoto University Faculty of Medicine, Kyoto, Japan). The breeding scheme to generate triple-homozygous mice is detailed in Supplemental Methods and illustrated in Supplemental Figure 1A.

All mouse strains and genotypes generated for these studies were congenic withC57BL/6J. Both male and female animals (aged 8–14 weeks) were utilized in TAM induction protocols. All mice were housed under pathogen-free conditions in an Association for Assessment and Accreditation of Laboratory Animal Care–approved (AAALAC-approved) barrier facility at the Perelman School of Medicine, University of Pennsylvania.

### Reagents and materials

Cytological stains used were Diff-Quik (Thermo Fisher Scientific) and Giemsa (GS500; MilliporeSigma). TAM (nonpharmaceutical grade) was purchased from MilliporeSigma. Nintedanib was purchased from Cayman Chemical. OBE022 (35, 41) and BAY6872 (36) were manufactured for Calico LLC by Abbvie Inc. Except where noted, all other reagents were electrophoretic or immunological grade and purchased from commercial companies as noted.

### Antibodies

Antibodies used for flow cytometry and FACS were obtained from commercial sources (Supplemental Table 1).

### Lung histology

Whole lungs were fixed by tracheal instillation of 10% neutral buffered formalin (MilliporeSigma) at a constant pressure of 25 cm H_2_O. In total, 6 μM sections were stained with H&E or Masson’s trichrome stains by the Pathology Core Laboratory of Children’s Hospital of Philadelphia. Slides were scanned using an Aperio ScanScope Model: CS2 (Leica) at 40× magnification; representative areas were captured and exported as TIF files and processed in Adobe Illustrator.

### PSR staining

Staining of lung sections for fibrillar collagen was performed using the PSR Stain Kit following the manufacturer’s instructions (Polysciences). Digital morphometric measurements were performed on multiple lobes and multiple levels, with 10 random peripheral lung images devoid of large airways per slide analyzed at a final magnification of 100× using ImageJ (NIH) as published (34, 45, 46). The mean area of each lung field in each section staining for PSR was calculated and expressed as a percentage of total section area as adapted from Henderson et al. (47).

### BALF collection and processing

BALF collected from mice using sequential lavages of lungs with 5 × 1 mL aliquots of sterile saline was processed for analysis as described (48, 49). Cell pellets obtained by centrifuging BALF samples at 400*g* for 6 minutes at 4°C were resuspended in 1 mL of PBS, and total cell counts were determined using a NucleoCounter (New Brunswick Scientific). Differential cell counts were determined manually from BALF cytospins stained with modified Giemsa (Sigma-Aldrich, GS500) to identify macrophages, lymphocytes, eosinophils, and neutrophils. Total protein content of cell-free BALF was determined using the DC Protein Assay Kit (Bio-Rad, 5000111) with BSA as a standard according to the manufacturer’s instructions.

### Determination of BALF-soluble collagen content and TGF-β1 concentration

Total acid soluble collagen content in cell-free BALF was determined using the Sircol assay kit (Biocolor) according to the manufacturer’s instructions. Total TGF-β concentration (latent and active) in the cell-free BALF was calculated using Mouse TGF-β1 DuoSet Elisa (R&D Systems, DY1679-05) according to the manufacturer’s instructions.

### RNA isolation and qPCR

RNA was extracted from homogenized lung or isolated fibroblasts using RNeasy Mini Kit (Qiagen) following the manufacturer’s protocol. The concentration and quality of extracted RNA from the lung tissues were measured using NanoDrop One (Thermo Fisher Scientific) and reverse transcribed into cDNA using either Taqman Reverse Transcription Reagents (Applied Biosystems) or Verso cDNA Synthesis Kit (Thermo Fisher Scientific).

qPCR for whole-lung *Col1a1*, *Col1a2*, and *Ptgfr* was performed using TaqMan Gene Expression Assays in an Applied Biosystems ViiA 7 real-time PCR system with a 384-well plate. Results were normalized to *Hprt*. Whole lung *Sftpc* as well as fibroblast lysate *Ces1d*, *Col13a1*, *Col14a1*, *Slc7a10*, *Pi16*, *Ebf1*, *Col14a1*, *Sfrp1*, *Hp*, *Tgfb1*, and *Cthrc1* were measured by qPCR on a QuantStudio 7 Flex Real-Time PCR System, with results normalized to *18S* and *Actb* RNA. Primer sequences for all mouse genes are listed in Supplemental Table 2.

### scRNA-Seq and analysis of lung cell populations

To capture representative proportions of all major parenchymal and immune cell populations, we profiled 94,258 cells from the model; single-cell suspensions were prepared by physical and enzymatic dissociation, followed by MACS by LS columns (Miltenyi Biotec, 130-042-401) with CD45^+^ cell removal using CD45 micobeads (Miltenyi Biotec, 130-052-301) followed by followed by “spike-back” of immune cells (~20% of final suspensions) with biological replicates for each time point loaded onto individual GemCode instrument (10× Genomics; 2 for each of the time points). Single-cell barcoded droplets were produced using 10X Single Cell 3′ v3 chemistry. Libraries generated were sequenced using the HiSeq Rapid SBS kit, and the resulting libraries were sequenced across the 2 lanes of an Illumina HiSeq2500 instrument in a high-output mode. scRNA-Seq reads were aligned to mouse genome (mm10/GRCm38) using STARSolo (version 2.7.5b). After initial quality control and processing, we analyzed the scRNA-Seq data using the Scanpy pipeline (50). Genes expressed in fewer than 3 cells were removed, and cells with fewer than 200 genes and a mitochondrial fraction of less than 20% were excluded. Counts were log normalized using scanpy.pp.normalize_per_cell (counts_per_cell_after=1x10^4^), followed by scanpy.pp.log1p. To integrate data from multiple samples, we used Scvi-tools (51). We applied scvi.model.SCVI.setup_anndata() to establish the model parameters for integration, including: layer, categorical_covariate_keys and continuous_covariate_keys. We then performed a principal component analysis (PCA) and generated a K-nearest neighbor (KNN) graph using scanpy.pp.neighbors with n_neighbors=15. The resulting KNN graph was used to perform Uniform Manifold Approximation and Projection (UMAP) dimension reduction to visualize the cells in 2 dimensions using scanpy.tl.umap(). Clustering was performed using the Leiden algorithm with scanpy.tl.leiden (52). We identified cell populations using known canonical marker genes or by assessing cluster-defining genes based on differential expressions. Additionally, we performed linear trajectory inference on the UMAP reduction using scFates (53) with the Adventitial fibroblast cluster and the Alveolar fibroblast cluster as the starting poinst and without assigned endpoints. Finally, we performed gene ontology analysis for enriched biological processes using GSEApy (54) based on differentially enriched genes between the groups.

### Multichannel flow cytometry for identification of lung cell populations

Flow cytometry was performed as we described (34, 38, 46, 49). Blood-free perfused lungs were digested in Phosphate Buffered Saline (Mg and Ca free) with 2 mg/mL Collagenase Type I (Thermo Fisher Scientific, catalog 17100017) and 50 units of DNase (MilliporeSigma, catalog D5025), passed through 70 μm nylon mesh to obtain single-cell suspensions, and then processed with ACK Lysis Buffer (Thermo Fisher Scientific). Cell pellets collected by centrifugation (800*g* for 5 minutes at 4°C) were resuspended in PBS with 0.1% sodium azide, and aliquots were removed for determination of cell number using a NucleoCounter (New Brunswick Scientific). Cells were incubated with antibody mixtures (or isotype controls) and conjugated viability dye (Supplemental Table 1).

Stained cells were analyzed with an LSR Fortessa (BD Biosciences). Cell populations were defined, gated, and analyzed with FlowJo software. Immune populations were identified by forward and side scatter, followed by doublet discrimination of CD45^+^ viable cells and a sorting strategy modified from Misharin et al. (55). Fibroblast subpopulations were isolated using published strategies (19–21). Mesenchyme was defined as Dapi^+^CD45^–^CD31^–^CD326^–^. Alveolar fibroblasts were further defined as mesenchyme PDGFRA^+^MCAM^–^Sca1^–^, while adventitial fibroblasts were defined as mesenchyme PDGFRa^+^MCAM^–^Sca1^+^. Confirmation of marker gene expression consistent with target cell populations was determined by qPCR.

### Isolation, in vitro culture, and challenge of mesenchymal populations

Generation of single-cell suspension and processing was performed as above. Mesenchymal populations were stained using a modified sorting strategy from Tsukui et al. (20) with a BD FACSAria II (BD Biosciences) by the Flow Cytometry Core at the University of Pennsylvania. Collected cell populations were immediately seeded in tissue culture at a concentration of 2 × 10^5^ cells per 1.9 cm^2^ in DMEM containing 5% FBS and 10 ng/mL TGF-β (BioLegend, catalog 763102) or 500 nM PGF (Cayman, catalog 16010).

### Measurement of urinary prostanoids

Urinary prostanoid metabolites were measured by liquid chromatography–mass spectrometry as described (56). Such measurements provide a noninvasive, time-integrated measurement of systemic prostanoid biosynthesis (57). Briefly, mouse urine samples were collected using metabolic cages over an 8-hour period (9 a.m. to 5 p.m.). Systemic production of PGI_2_, PGE_2_, PGD_2_, and TxA_2_ was determined by quantifying their major urinary metabolites — 2, 3-dinor 6-keto PGF_1α_ (PGIM); 7-hydroxy-5, 11-diketotetranorprostane-1, 16-dioic acid (PGEM); 11,15-dioxo-9_α_-hydroxy-2, 3, 4, 5-tetranorprostan-1, 20-dioic acid (tetranor PGDM), and 2,3-dinor TxB_2_ (TxM), respectively. Results were normalized with urinary creatinine.

### Statistics

All data are presented with dot plots and group mean ± SEM, unless otherwise indicated. Statistical analyses were performed with GraphPad Prism. Student’s *t* test (1 or 2 tailed, as appropriate) were used for 2 groups. Multiple comparisons were done by 1-way ANOVA, which was performed with post hoc testing as indicated; survival analyses was performed using Kaplan-Meier with Mantel Cox correction. In all cases, statistical significance was considered at *P* ≤ 0.05.

### Data availability

RNA-Seq data generated in this study are deposited in Gene Expression Omnibus (GSE234604). All other data re provided in this article can be accessed in the Supporting Data Values file.

### Study approval

Mice housed in pathogen-free facilities were subjected to experimental protocols approved by the IACUC of the Perelman School of Medicine at the University of Pennsylvania.

## Author contributions

MFB and GAF developed the concept. MFB, LRR, SYT, TQC, and GAF designed the experiments. SYT and YT performed in vivo animal experiments. LRR, SYT, SI, KC, CHC, TTD, and AM performed in vitro experiments and endpoint analyses for in vivo studies. USD and SG performed mass spectrometric analyses of eicosanoids. WRB, AB, and CC performed bioinformatic analysis. AM performed flow cytometry. TQC, SYT, AM, LRR, and MFB analyzed data, generated figures, and interpreted results. LRR and MFB drafted the manuscript. GAF, MFB, and LRR edited the manuscript. All authors reviewed and approved the final version prior to submission.

## Supplementary Material

Supplemental data

Supporting data values

## Version 1 11/07/2023

In-Press Preview

## Version 2 12/22/2023

Electronic publication
