# Supplementary material for: PGF2α signaling drives fibrotic remodeling and fibroblast population dynamics in mice
Source: JCI Insight. 2023 Dec 22;8(24):e172977. doi: 10.1172/jci.insight.172977 (PMC10807712; doi:10.1172/jci.insight.172977)
Supplement: Supplemental data [file jciinsight-8-172977-s114.pdf]

SUPPLEMENTAL METHODS and DATA

PGF2α Signaling Drives Fibrotic Remodeling And Fibroblast Population Dynamics In Mice

Luis R. Rodriguez[1,2 *], Soon Yew Tang[3 *], Willy Roque Barboza[1,2 *], Aditi Murthy[1, 2], Yaniv Tomer[1, 2], Tian-Quan Cai[4], Swati Iyer[1,2], Katrina Chavez[1,2], Ujjalkumar Subhash Das[3], Soumita Ghosh[3], Charlotte Cooper[1,2], Thalia Dimopoulos[1,2], Apoorva Babu[2], Caitlin Connelly[4], Garret A FitzGerald[3 ¶], and Michael F. Beers[1, 2 ¶]

[1] Pulmonary and Critical Care Division Department of Medicine; Perelman School of Medicine at the University of Pennsylvania; Philadelphia, PA 19104
[2] PENN-CHOP Lung Biology Institute; Perelman School of Medicine at the University of Pennsylvania; Philadelphia, PA 19104
[3] Institute for Translational Medicine and Therapeutics; Department of Systems Pharmacology and Translational Therapeutics; Perelman School of Medicine at the University of Pennsylvania; Philadelphia, PA 19104
[4] Calico Life Sciences LLC, South San Francisco, CA 94080.

[*] **These authors provided equal contribution to this manuscript**

Running Title: *Prostaglandin F2α Signaling in Lung Fibrosis*

¶ Correspondence should be addressed to:

Michael F. Beers, M.D.
Pulmonary and Critical Care Division
Perelman School of Medicine at The University of Pennsylvania
Edward J Stemmler Hall Suite 216
3450 Hamilton Walk
Philadelphia, Pennsylvania 19104-5159
e-mail: **mfbeers@pennmedicine.upenn.edu**

Garret FitzGerald, M.D.
Institute for Translational Medicine and Therapeutics
Department of Systems Pharmacology and Translational Therapeutics
Perelman School of Medicine at the University of Pennsylvania
Room 10-122, Smilow Center for Translational Research (SCTR)
34th and Civic Center Boulevard
Building 421
Philadelphia, PA 19104-5158
e-mail: **garret@upenn.edu**

[†] Albert M. Rose Established Investigator of the Pulmonary Fibrosis Foundation

Funding: NIH UO1 HL119436 (MFB), VA Merit Review 2 I01 BX001176 (MFB) a sponsored research agreement from Calico Life Sciences, LLC (GAF), NIH F32 HL160011 (LR) and a Pulmonary Fibrosis Foundation Scholars Award (LR).

Conflict of Interest: GAF is an advisor to Calico Life sciences. Otherwise, the authors declare that no conflicts of interest exist.

SUPPLEMENTAL METHODS

Sftpc^{I73T} Targeting Vector and Recombineering strategy for generation of SP-C^{I73T} founder line

The *Sftpc^{I73T}* targeting vector was commercially produced by Gene Bridges, GmbH (Heidelberg, Germany) using a BAC-encoded fragment of interest from a murine C57BL/6 strain. Synthesis of a genomic DNA fragment carrying a hemagglutinin tag (HA) tag in Exon1 (the NH_2 terminus), an Ile for Thr substitution in Exon 3a, and the *FRT-PGK-gb2-Neo/km-FRT* cassette in Intron 1 was performed. *FRT-PGK-gb2-Neo/km-FRT* (herein termed "*PGK-Neo"* cassette) encodes for *gb2* driven kanamycin resistance in *E. coli* and mouse phosphoglucokinase promoter (PGK) driven expression of neomycin resistance in mammalian cells and is flanked by short flippase recognition target (FRT) sites The modified DNA fragment carrying the point mutation was then inserted into the *Sftpc* locus of the corresponding Zeocin/*Sftpc* BAC by one Red/ET step (triple recombination) and the resultant BAC encoded product subcloned into a minimal pMV vector. The functional regions (FRT, point mutation, restriction sites, and resistance cassette) were verified by sequencing.

ES cell electroporation of this targeting vector, clone selection, and microinjection of targeted ES clones into blastocysts of Balb/c mice were performed by University of Pennsylvania Transgenic Core Facility. F_0 offspring with a high degree of coat chimerism were then crossed with C57/Bl6 6J mice and germ line transmission of the recombineered allele screened by color, PCR, and Southern blot hybridization. The resultant SP-C^{I73T} founder line was designated "SP-C$^{I73T\text{-}Neo}$".

Tamoxifen inducible model of Sftpc^{I73T} expression- For inducible removal of PGK-*Neo* from *Sftpc$^{I73T\text{-}Neo}$* alleles, a Flp-O recombinase "deleter line" which contained an optimized FLPe recombinase variant (Flp-O) under control of the mutated ER_2 receptor knocked into the Rosa26 locus (Strain *B6N.129S6(Cg)-Gt(ROSA)26Sortm3 (CAG-FLPo/ ERT2)Alj/J*) was purchased from The Jackson Laboratory (Bar Harbor, ME)((Stock # 01906) and crossed with the HA-SP-C$^{I73T\text{-}Neo}$ line. Genotyping of the resultant line, termed **"IER-SP-C^{I73T}"**, was performed as described below.

Generation of F Prostanoid receptor (FPr)- deficient IER-Sftpc^{I73T} mouse model : Triple homozygous (IER-SP-C$^{I73T/I73T}$ FlpO$^{+/+}$ Fpr$^{-/-}$) mice lacking Ptgfr were generated by initially crossing male Sftpc$^{I73T/I73T}$ / Flp$^{+/+}$ mice with female FPr$^{+/-}$ mice. The presence of SP-C^{I73T}, FlpO, and FPr alleles were assessed by

PCR analyses. Multiple intercrosses resulted in generation of $Sftpc^{I73T/I73T}/Flp^{+/+}/Fpr^{-/-}$ ("FPr knockouts"), $Sftpc^{I73T/I73T}/Flp^{+/+}/Fpr^{+/-}$ ("FPr heterozygotes") and $Sftpc^{I73T/I73T}$ / $Flp^{+/+}$/ $Fpr^{+/+}$ ("FPr controls"). Additional controls included a FPr deficient, Sftpc wild-type strain (IER-$Sftpc^{WT/WT}/Flp^{+/+}/Fpr^{-/-}$).

PCR based genotyping of mice

DNA was extracted from tail snips obtained from pre-weaned mouse pups and then used as template for detection of the following alleles by polymerase chain reaction (PCR):

$Sftpc^{Wild\text{-}type}$, $Sftpc^{I73T}$, and $SP\text{-}C^{I73T\text{-}Neo}$ - Multiplex PCR was done using a common reverse primer 3' downstream of the PGK-Neo insertion site, with alternate forward priming sites located upstream of or within the PGK-Neo cassette. Amplification was performed using Platinum Taq (Invitrogen), with the following primer sequences:

SP-C Fwd: TCACCCCTGTCCTCTCTGTC
PGK Fwd: TGGATGTGGAATGTGTGCGA
SP-C Rev: CCCAACTACATGGTGGTGCTA

The thermal cycling conditions were: 95°C for 3 minutes; 37 cycles of 95°C (30sec), 64°C (30 sec), 72°C (60 sec). For *$Sftpc^{Wld\text{-}type}$* this results in amplification of a 433 BP product. For *$Sftpc^{I73T}$* alleles post-excision, 113 base pairs (including the FRT and HA sites) are added, resulting in a 446 bp band. For alleles pre-excision (*$SP\text{-}C^{I73T\text{-}Neo}$*), an alternate forward priming site within the PGK promoter permits preferential amplification of a 271 bp band.

R26Flp-O ER - Multiplex PCR was done using a common forward primer and two reverse primers (as described by Jackson Laboratories, Inc.). Amplification was performed using Platinum Taq (Invitrogen), with the following primer sequences:

Common Forward (oIMR8545): AAAGTCGCTCTGAGTTGTTAT
I73T Mutant Reverse (10507): TTATGTAACGCGGAACTCCA
Wild type Reverse (oIMR8546): GGAGCGGGAGAAATGGATATG

The thermal cycling conditions used were: 95°C for 3 minutes; 10 cycles of 95°C (30sec) 65 - 0.5 °C/cycle (30sec), 68°C (60sec); 28 cycles of 95°C(30 sec), 60°C (30sec) 72°C (60 sec). These conditions result in amplification of a 603 bp band (Flp-O negative) and a 309 bp band (Flp-O positive).

Ptgfr Status - The thermal cycling conditions were: 95°C for 5 minutes; 35 cycles of 94°C (30sec), 65°C (30 sec), 75°C (60 sec). For *Ptgfr*$^{Wld\text{-}type}$ this results in amplification of a 700 BP product. For *Ptgfr*^{KO} alleles this results in a 450 bp band. Amplification was performed using the following primers:

FP1: GCC CAT CCT TGG ACA CCG AGA TTA TCA A-3'
FP2: AGA GTC GGC AAG CTG TGA CTT CGT CTT-3'
FP3: TGA TAT TGC TGA AGA GCT TGG CGG CGA A-3'

Determination of FPr inhibitor Efficacy in A Bleomycin Induced Mouse Lung Fibrosis Model

OBE-022 and BAY6672 were evaluated in a bleomycin (BLM) induced lung fibrosis model performed by HD Biosciences, Ltd. Shanghai, PR China. For each compound, 85 male C57/Bl6 male mice were randomly divided into 5 groups: 1) Group 1 (17 mice): Sham group, mice were administered with PBS (i.t) and received vehicle (0.5% [w/v] HPMC and 0.02% [v/v] Polysorbate 80) in Water (p.o., QD); 2) Group 2 (17 mice): Model group, mice were administered with BLM (0.66mg/kg, i.t) and received vehicle (0.5% [w/v] HPMC and 0.02% [v/v] Polysorbate 80) in Water (p.o., QD); 3) Group 3 (17 mice): OBE022 (100mpk) or BAY6672 (30 or 100 mpk). Mice were administered with BLM (0.66mg/kg, i.t) and received OBE022 or (100mpk, p.o., BID or BAY6872 (30 or 100 mpk)); 4) Group 4 (17 mice): Calico-006, 300mpk, mice were administered with BLM (0.66mg/kg, i.t) and received Calico-006 (300mpk, p.o., BID); 5) Group 5(17 mice): Nintedanib 60mpk, mice were administered with BLM (0.66mg/kg, i.t) and received Nintedanib (60mpk, p.o., QD). The mice were administered with BLM (0.66mg/kg, i.t.) on day 1. After the onset of injury, the mice were randomized with dosing beginning on day 5 after BLM injection. Compound treatment occurred for 17 days of an entire study period lasting 21 days.

End point assays included (i) Total body weight; (ii) BALF cell counts and cytological differentials; BALF soluble collagen content using Sircol assay (ii) Histopathological examination for collagen deposition and inflammation quantification (iv) IHC analysis (staining and quantification) of α-SMA. Histopathological evaluation of lung using Masson Trichrome Staining and fibrosis was evaluated by Modified Ashcroft scoring. Whole slide scanning and image analysis was performed to quantify α-SMA in the fibrotic lung tissues by positive area.

SUPPLEMENTAL FIGURES WITH LEGENDS

Supplemental Figure S1. (A) Schematic describing breeding strategy to generate IER-*Sftpc^{I73T}*/ *Ptgfr$^{+/+}$*, IER-*Sftpc^{I73T}*/ *Ptgfr$^{-/-}$*, and IER-*Sftpc^{I73T}*/ *Ptgfr$^{-/-}$*. **(B)** Quantification of urinary prostanoids in urine of IER-*Sftpc^{I73T}*/ *Ptgfr$^{+/+}$* (n=22) and IER-*Sftpc^{I73T}*/ *Ptgfr$^{-/-}$* (n=13) post tamoxifen induction through the progression of the model. Increases in Prostacyclin (PGI), Prostaglandin E (PGE), Prostaglandin D (PGD), and Thromboxane (Tx) can be observed during the first 7 days with PGI and Tx demonstrating a continuous rise through the 21- and 14-day timepoint respectively. Elevated levels of all four prostanoids are sustained through the fibrotic time points. In contrast urinary creatine is decreased throughout the model. Statistical testing was performed using mixed effects modeling with *$p < 0.05$

Supplemental Figure S2. Alternative Protocols for tamoxifen in *Sftpc^{I73T}* do not alter model outcomes. (A) Schematic describing three approaches to administer tamoxifen in IER-*Sftpc^{I73T}* mice: Single intraperitoneal delivery (n=13) , split intraperitoneal delivery on day 0 and 3 (n=14), or split gavage on day 0 and 4 (n=12). **(B)** Weight loss nadir in IER-*Sftpc^{I73T}* mice is similar across all administration protocols with a more gradual progression observed using the split dose oral gavage protocol. **(B)** Expression of *Sftpc* as measured by qPCR is comparable in all three protocols. **(D)** Total cell count in BALF 28 days after first tamoxifen induction is comparable across all three protocols. Statistical testing was performed using ordinary one-way ANOVA.

Supplemental Figure S3. Effect of nintedanib treatment on spontaneous fibrotic lung remodeling following oral tamoxifen induction of Sftpc^{I73T}expression*.* (A) Schematic of protocol used for tamoxifen induction of IER-*Sftpc*^{I73T} mice and nintedanib treatment. **(B)** Weight loss in induced *Sftpc*^{I73T} mice receiving nintedanib (n=13) or vehicle (n=13) at Day 12. **(C-E)** BALF collected from IER-*Sftpc*^{I73T} mice 28 days post tamoxifen induction demonstrates increased cell count, total protein, and soluble collagen (Sircol™ Fibrillar collagen assay) as compared to C57B6 (n=7) controls. Nintedanib partially mitigates these metrics with a significant decrease in BALF total protein after intervention. **(F-G)** Lung compliance is significantly reduced in IER-*Sftpc*^{I73T} mice with a nonsignificant partial mitigation observed after nintedanib treatment. **(H)** Quantification of fibular collagen in histological sections (reported as % PSR Stained Area) from *Sftpc*^{I73T} lungs 28 days post-tamoxifen demonstrates a significant reduction in collagen deposition after nintedanib intervention. **(I-J)** Representative histology from IER-*Sftpc*^{I73T} mice 28 days post tamoxifen induction Statistical testing was performed using ordinary one-way ANOVA with *$p < 0.05$ ** $p<0.005$ *** $p<0.0005$.

Supplemental Figure S4. Pharmacological Inhibition of FPr Signaling Decreases Fibrotic Endpoints After Bleomycin Injury. (A) Schematic of pharmacological intervention strategy used for OBE-222 and BAY-6672. Treatment was started on Day 6 and maintained through day 22 with final collection occurring 2 hours post final dose administration. **(B)** Endpoint analysis of OBE-022 treatment compared to nintedanib intervention. All groups were made up of n=17 animals. No significant differences were observed in body weight across all interventions. Cell count and soluble collage in BALF after bleomycin challenge was significantly reduced in all intervention protocols as compared to vehicle controls. Histological scoring applying modified Ashcroft Score resulted in a significantly lower score after nintedanib intervention. **(C)** Histological scoring of BAY-6672 (alternative identifier A422), nintedanib, and combined interventions. All groups were made up of n=12 animals. Modified Ashcroft scoring demonstrates mitigation of fibrotic severity post bleomycin injury in all interventions with no significant difference when nintedanib is combined with BAY-6672. This trend is also observed in collagen and smooth muscle actin quantification. Statistical analysis was performed using student's one-way ANOVA *$p < 0.05$ ** $p<0.005$ *** $p<0.0005$.

Supplemental Figure S5. *Ptgfr* Deficiency Does not Alter Tissue Lymphocyte Populations in IER-*Sftpc^{I73T}*. (A) Gating strategy used to quantify B-cells, CD4 T cells, and CD8 T cells in single cell suspensions derived from murine lungs. **(B)** No significant difference in tissue lymphocyte percentages was observed between IER-*Sftpc^{I73T}*/ *Ptgfr$^{+/+}$* (n=5) and IER-*Sftpc^{I73T}*/ *Ptgfr$^{-/-}$*(n=5) mice **(C)** Gating strategy used to quantify neutrophils, eosinophils, and Ly6c hi monocytes in single cell suspensions derived from murine lungs. Statistical testing was performed using ordinary one-way ANOVA.

Supplemental Figure S6. Single Cell Sample Integration in IER-*Sftpc*^{I73T}. (A) UMAP projection of integrated data set identifies the four major cell compartments in murine lungs **(B)** UMAP projection of integrated data set with color designations for each timepoint and genotype. **(C-E)** UMAP projections of integrated divided by individual samples with single color designations

Supplemental Figure S7. Cell Type Identification in Integrated IER-*Sftpc*^{I73T} Single Cell Data (A) UMAP projections of integrated data sets highlighting cluster genes used to identify mesenchymal subsets. **(B)** UMAP projections of integrated data sets highlighting cluster genes used to identify natural killer cells. **(C)** UMAP projections of integrated data sets highlighting cluster genes used to identify endothelial cells. **(D)** UMAP projections of integrated data sets highlighting cluster genes used to identify dendritic cells. **(E)** UMAP projections of integrated data sets highlighting cluster genes used to identify epithelial cells. **(F)** UMAP projections of integrated data sets highlighting cluster genes used to identify neutrophils. **(G)** UMAP projections of integrated data sets highlighting cluster genes used to identify myeloid cells. **(H)** UMAP projections of integrated data sets highlighting cluster genes used to identify lymphocytes.

Supplemental Figure S8. Mesenchymal Cell Cluster Identification in *Sftpc*^{I73T} Integrated Single Cell Data Set. (A) UMAP projection of integrated mesenchymal compartment with color designations for each timepoint and genotype. **(B)** Alternative Marker genes for mesenchymal subsets graphed as a gradient dot plot. **(C)** UMAP projections of integrated mesenchyme highlighting cluster genes used to identify alveolar fibroblasts. **(D)** UMAP projections of integrated mesenchyme highlighting cluster genes used to identify fibrotic fibroblasts. **(E)** UMAP projections of integrated mesenchyme highlighting cluster genes used to identify transitional/inflammatory fibroblasts. **(F)** UMAP projections of integrated mesenchyme highlighting cluster genes used to identify adventitial fibroblasts. **(G)** UMAP projections of integrated mesenchyme highlighting cluster genes used to identify smooth muscle cells. **(H)** UMAP projections of integrated mesenchyme highlighting cluster genes used to identify pericytes. **(I)** UMAP projections of integrated mesenchyme highlighting cluster genes used to identify peribronchial fibroblasts. **(J)** UMAP projections of integrated mesenchyme highlighting cluster genes used to identify mesothelial cells.

Supplemental Figure S9. (A) Frequency plot of mesenchymal subsets within individual samples. (B) UMAP projections of *Pdgfra* positive mesenchymal clusters divided by timepoint and highlighting the expression of *Ptgfr*. (C) UMAP projection of integrated mesenchymal clusters divided by genotype and highlighting profibrotic markers (D) *Tgfb1* expression in mesenchyme and all lung compartments combined (mesenchyme, endothelial, epithelial, and immune) expressed as dot plots across conditions and timepoint. (E) *Tgfb1* expression in each major lung compartment throughout the time course of the model expressed as dot plots and split by genotype and timepoint**.**

Supplemental Figure S10. (A) Quantification of transitional cluster maker genes after 48-hour challenge in alveolar fibroblasts demonstrates that Fp (500 nM) does not induce the transitional state in alveolar fibroblasts. While TGFβ (10ng/ml) decreases expression of transitional state marker genes independent of *Ptgfr* status (B) Quantification of fibrotic cluster marker genes after 48-hour challenge confirms that TGFβ promotes entry of alveolar fibroblasts into the fibrotic state independent of FPr status. Ordinary one-way ANOVA statistical testing was performed with significance between groups denoted by * : *$p < 0.05$ ** $p<0.005$ *** $p<0.0005$. Statistical significance between treatment and media control denoted by & : & $p< 0.05$

SUPPLEMENTAL FIGURE LEGENDS

FIGURE S1

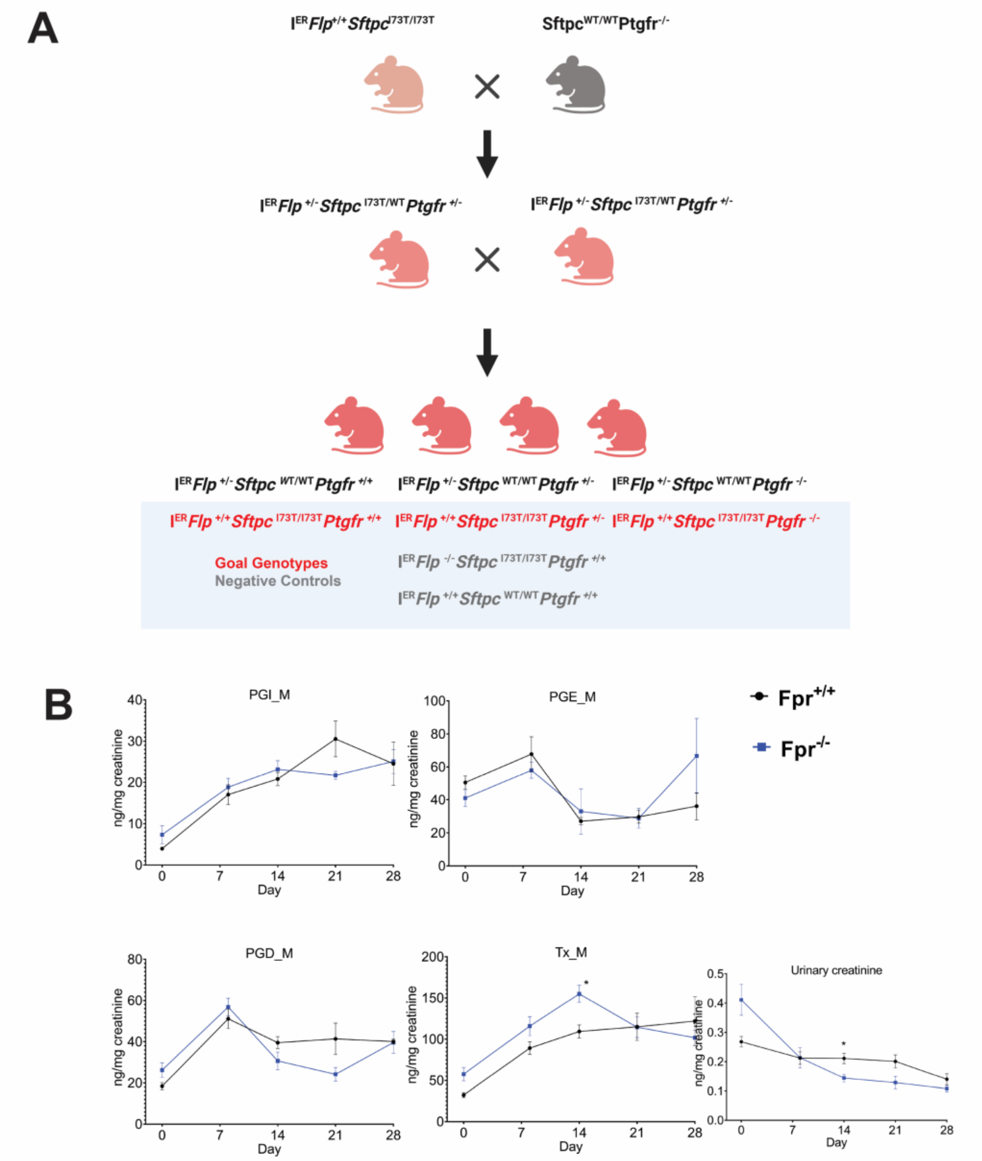

Supplemental Figure S1. (A) Schematic describing breeding strategy to generate IER-*Sftpc*^{I73T}/ *Ptgfr*$^{+/+}$, IER-*Sftpc*^{I73T}/ *Ptgfr*$^{-/-}$, and IER-*Sftpc*^{I73T}/ *Ptgfr*$^{-/-}$. **(B)** Quantification of urinary prostanoids in urine of IER-*Sftpc*^{I73T}/ *Ptgfr*$^{+/+}$ (n=22) and IER-*Sftpc*^{I73T}/ *Ptgfr*$^{-/-}$ (n=13) post tamoxifen induction through the progression of the model. Increases in Prostacyclin (PGI), Prostaglandin E (PGE), Prostaglandin D (PGD), and Thromboxane (Tx) can be observed during the first 7 days with PGI and Tx demonstrating a continuous rise through the 21- and 14-day timepoint respectively. Elevated levels of all four prostanoids are sustained through the fibrotic time points. In contrast urinary creatine is decreased throughout the model. Statistical testing was performed using mixed effects modeling with $^{*}p < 0.05$

FIGURE S2

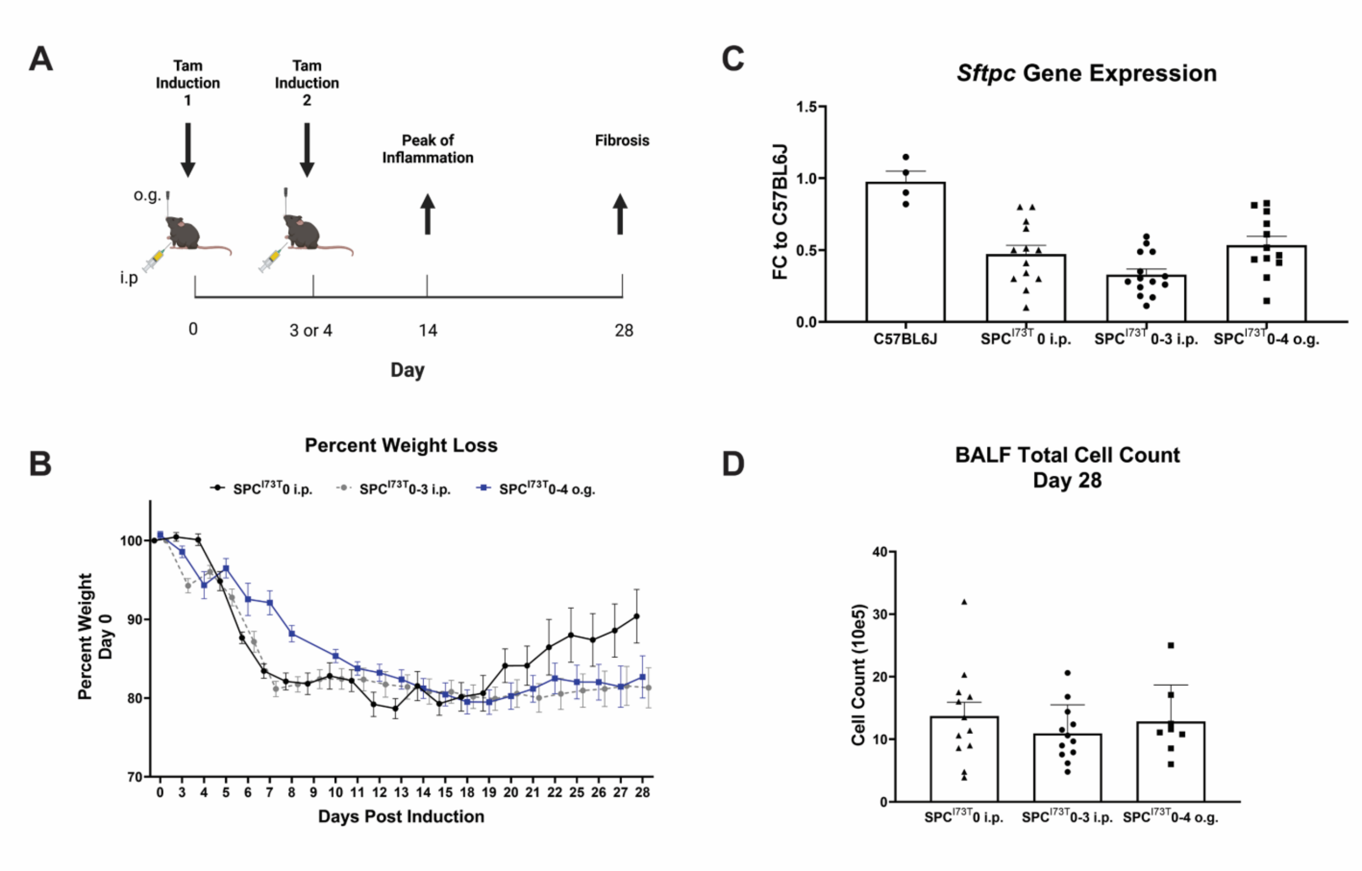

Supplemental Figure S2. Alternative Protocols for tamoxifen in *Sftpc*^{I73T} do not alter model outcomes. (A) Schematic describing three approaches to administer tamoxifen in IER-*Sftpc*^{I73T} mice: Single intraperitoneal delivery (n=13) , split intraperitoneal delivery on day 0 and 3 (n=14), or split gavage on day 0 and 4 (n=12). **(B)** Weight loss nadir in IER-*Sftpc*^{I73T} mice is similar across all administration protocols with a more gradual progression observed using the split dose oral gavage protocol. **(B)** Expression of *Sftpc* as measured by qPCR is comparable in all three protocols. **(D)** Total cell count in BALF 28 days after first tamoxifen induction is comparable across all three protocols. Statistical testing was performed using ordinary one-way ANOVA.

FIGURE S3

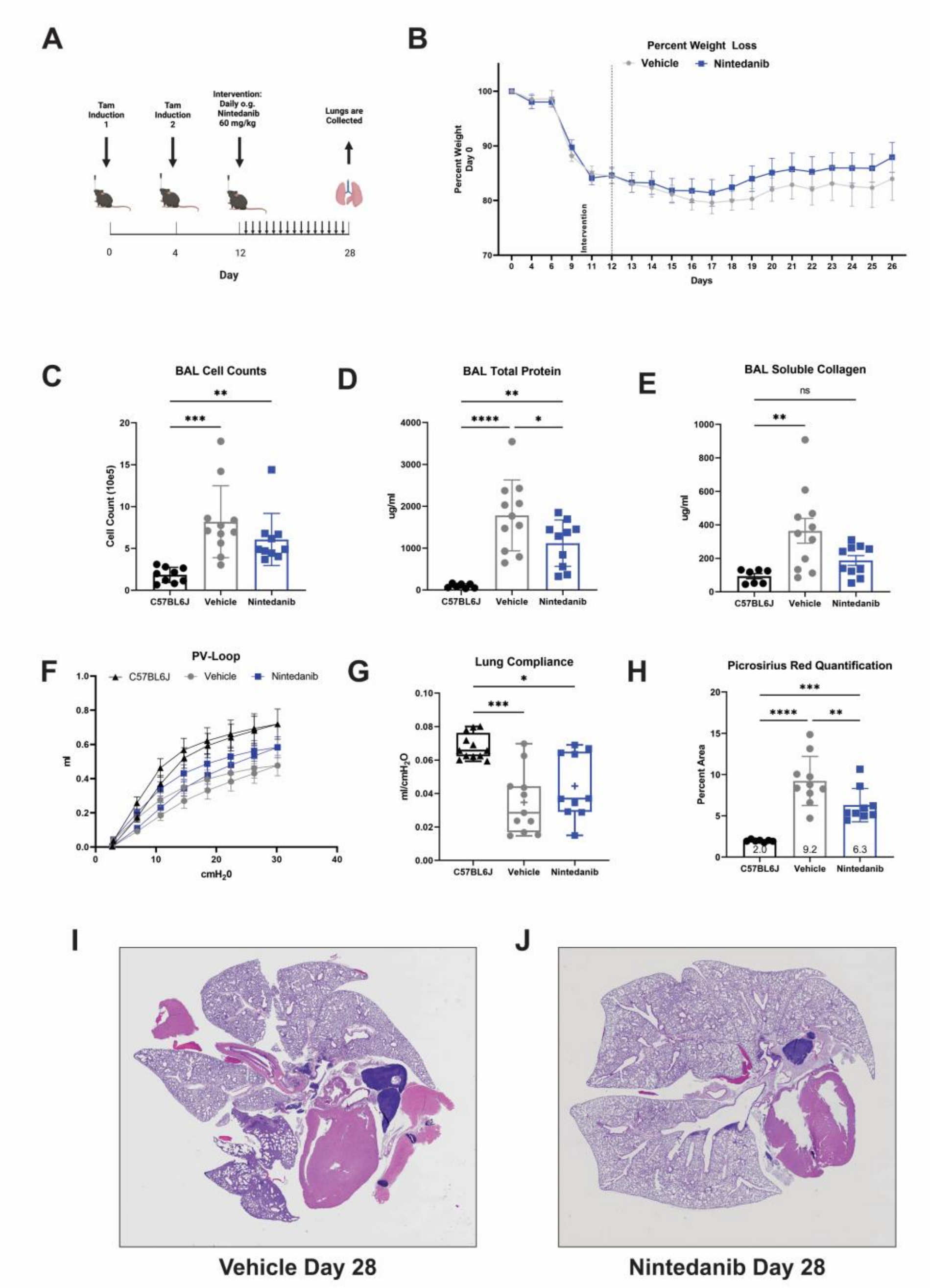

Supplemental Figure S3. Effect of nintedanib treatment on spontaneous fibrotic lung remodeling following oral tamoxifen induction of Sftpc^{I73T}expression*.* (A) Schematic of protocol used for tamoxifen induction of IER-*Sftpc*^{I73T} mice and nintedanib treatment. **(B)** Weight loss in induced *Sftpc*^{I73T} mice receiving nintedanib (n=13) or vehicle (n=13) at Day 12. **(C-E)** BALF collected from IER-*Sftpc*^{I73T} mice 28 days post tamoxifen induction demonstrates increased cell count, total protein, and soluble collagen (Sircol™ Fibrillar collagen assay) as compared to C57B6 (n=7) controls. Nintedanib partially mitigates these metrics with a significant decrease in BALF total protein after intervention. **(F-G)** Lung compliance is significantly reduced in IER-*Sftpc*^{I73T} mice with a nonsignificant partial mitigation observed after nintedanib treatment. **(H)** Quantification of fibular collagen in histological sections (reported as % PSR Stained Area) from *Sftpc*^{I73T} lungs 28 days post-tamoxifen demonstrates a significant reduction in collagen deposition after nintedanib intervention. **(I-J)** Representative histology from IER-*Sftpc*^{I73T} mice 28 days post tamoxifen induction Statistical testing was performed using ordinary one-way ANOVA with *p < 0.05 ** p<0.005 *** p<0.0005.

FIGURE S4

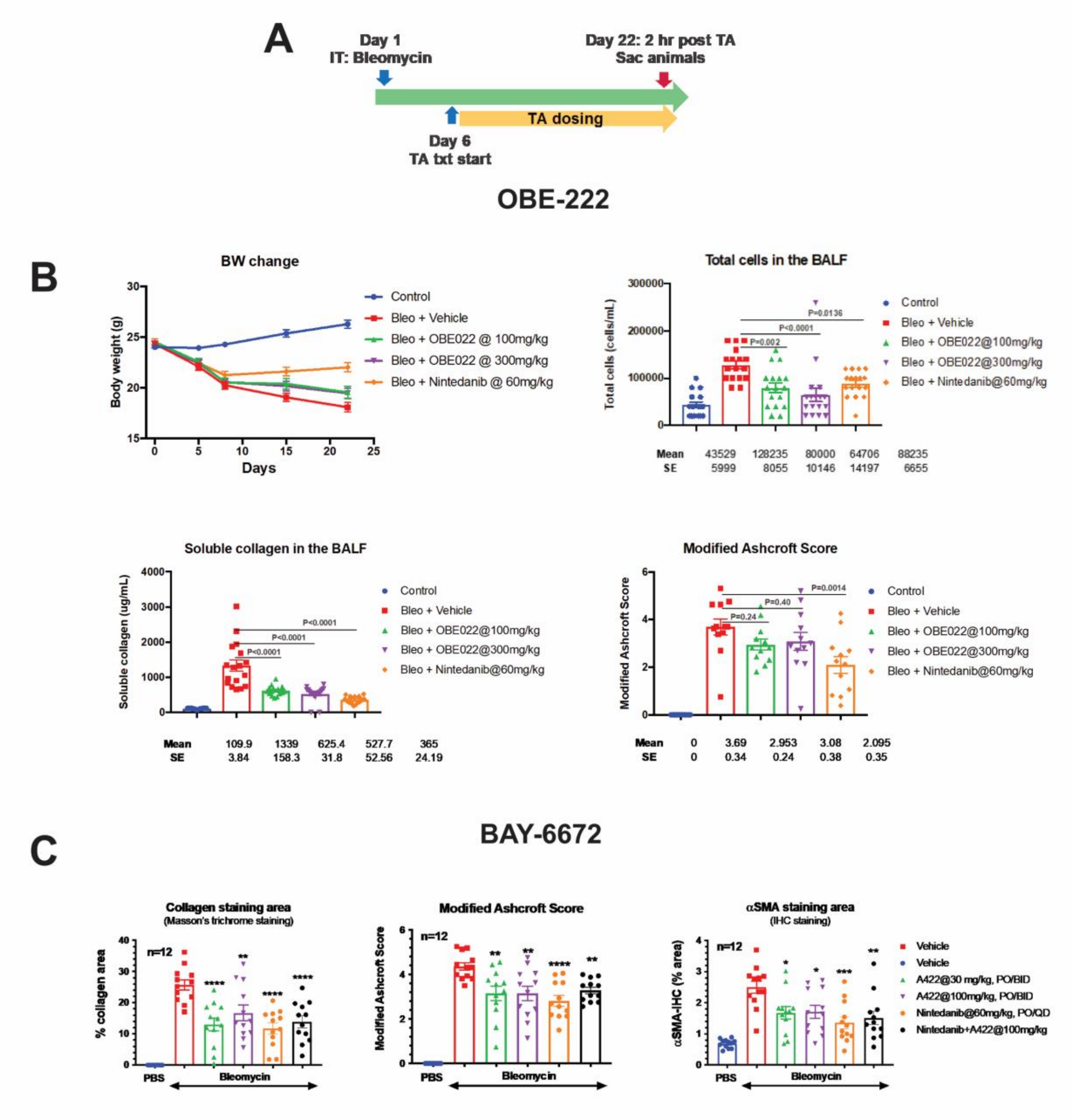

Supplemental Figure S4. Pharmacological Inhibition of FPr Signaling Decreases Fibrotic Endpoints After Bleomycin Injury. (A) Schematic of pharmacological intervention strategy used for OBE-222 and BAY-6672. Treatment was started on Day 6 and maintained through day 22 with final collection occurring 2 hours post final dose administration. **(B)** Endpoint analysis of OBE-022 treatment compared to nintedanib intervention. All groups were made up of n=17 animals. No significant differences were observed in body weight across all interventions. Cell count and soluble collage in BALF after bleomycin challenge was significantly reduced in all intervention protocols as compared to vehicle controls. Histological scoring applying modified Ashcroft Score resulted in a significantly lower score after nintedanib intervention. **(C)** Histological scoring of BAY-6672 (alternative identifier A422), nintedanib, and combined interventions. All groups were made up of n=12 animals. Modified Ashcroft scoring demonstrates mitigation of fibrotic severity post bleomycin injury in all interventions with no significant difference when nintedanib is combined with BAY-6672. This trend is also observed in collagen and smooth muscle actin quantification. Statistical analysis was performed using student's one-way ANOVA $^*p < 0.05$ $^{**}p<0.005$ $^{***}p<0.0005$.

FIGURE S5

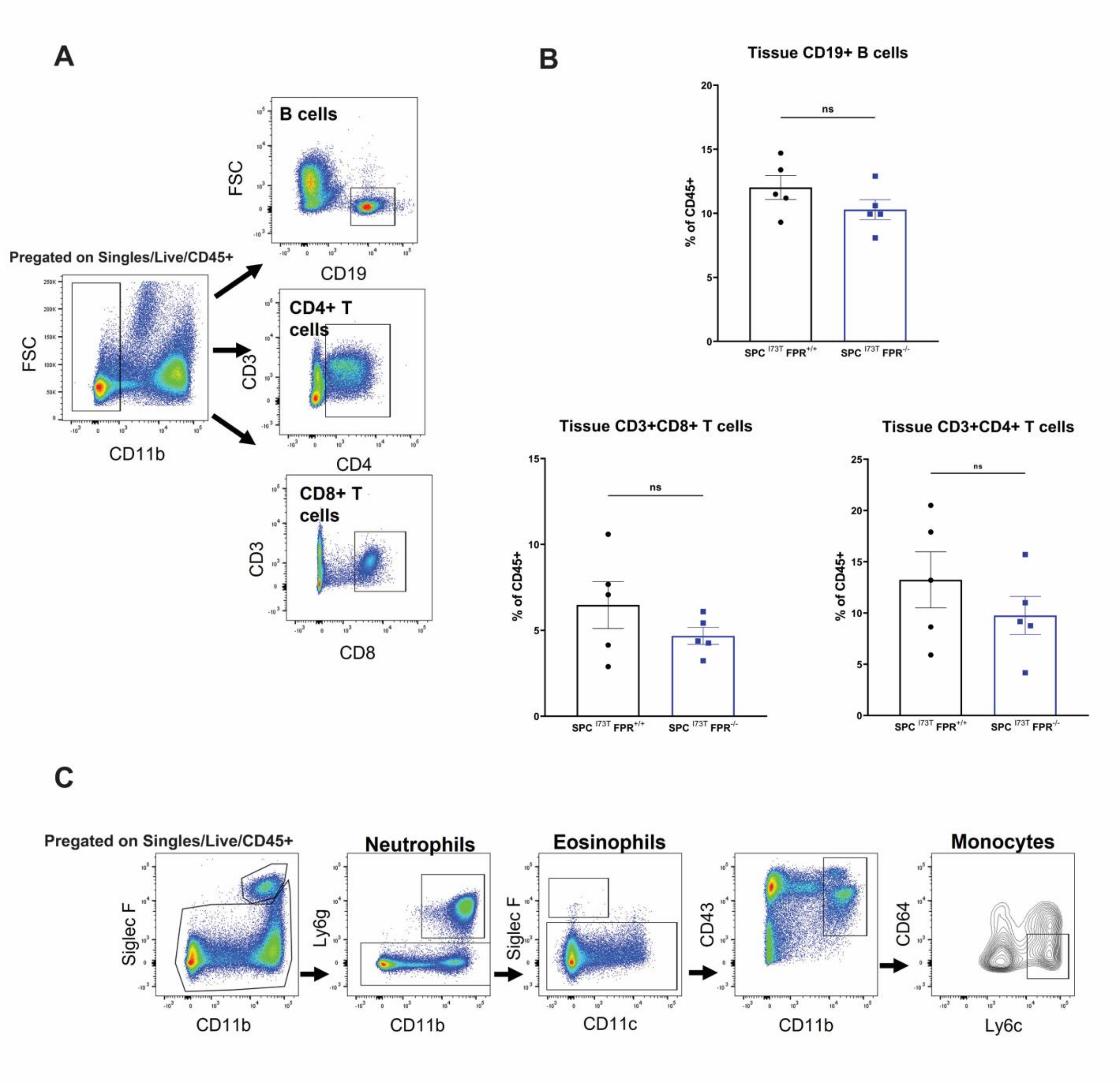

Supplemental Figure S5. *Ptgfr* Deficiency Does not Alter Tissue Lymphocyte Populations in IER-*Sftpc*^{I73T}. (A) Gating strategy used to quantify B-cells, CD4 T cells, and CD8 T cells in single cell suspensions derived from murine lungs. **(B)** No significant difference in tissue lymphocyte percentages was observed between IER-*Sftpc*^{I73T}/ *Ptgfr*$^{+/+}$ (n=5) and IER-*Sftpc*^{I73T}/ *Ptgfr*$^{-/-}$(n=5) mice **(C)** Gating strategy used to quantify neutrophils, eosinophils, and Ly6c hi monocytes in single cell suspensions derived from murine lungs. Statistical testing was performed using ordinary one-way ANOVA.

FIGURE S6

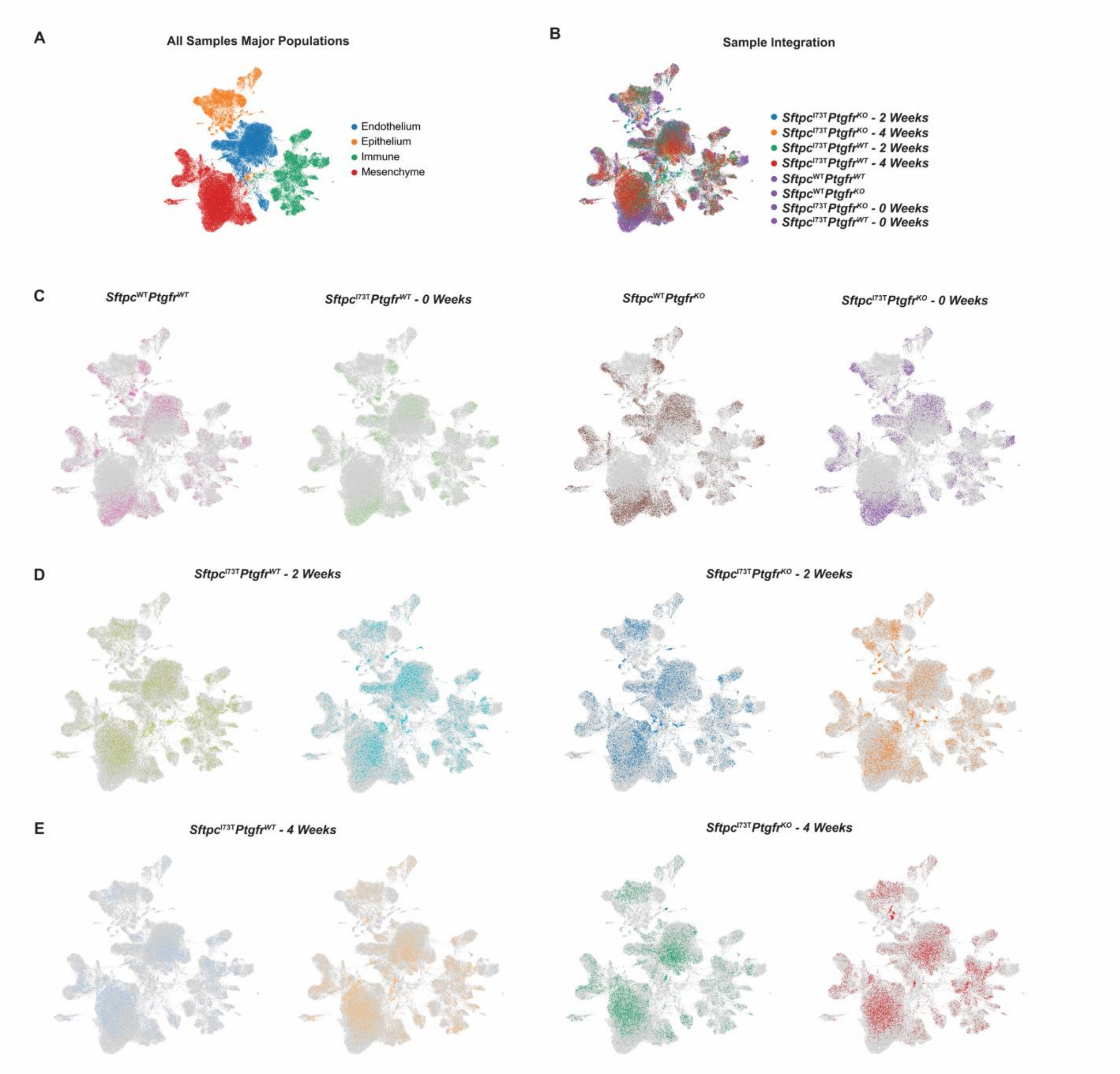

Supplemental Figure S6. Single Cell Sample Integration in IER-*Sftpc*^{I73T}. (A) UMAP projection of integrated data set identifies the four major cell compartments in murine lungs **(B)** UMAP projection of integrated data set with color designations for each timepoint and genotype. **(C-E)** UMAP projections of integrated divided by individual samples with single color designations

FIGURE S7

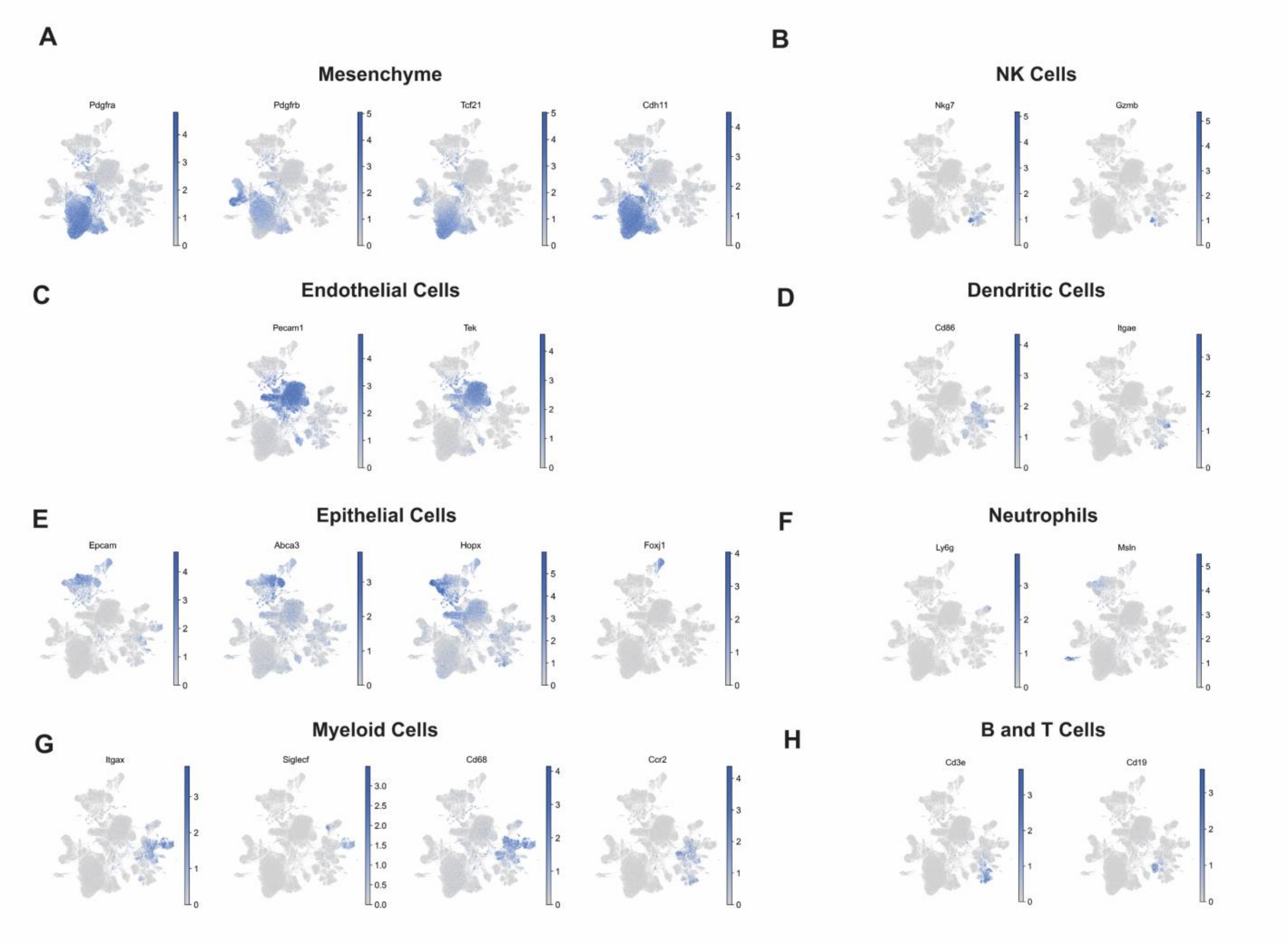

Supplemental Figure S7. Cell Type Identification in Integrated IER-*Sftpc*^{I73T} Single Cell Data (A) UMAP projections of integrated data sets highlighting cluster genes used to identify mesenchymal subsets. **(B)** UMAP projections of integrated data sets highlighting cluster genes used to identify natural killer cells. **(C)** UMAP projections of integrated data sets highlighting cluster genes used to identify endothelial cells. **(D)** UMAP projections of integrated data sets highlighting cluster genes used to identify dendritic cells. **(E)** UMAP projections of integrated data sets highlighting cluster genes used to identify epithelial cells. **(F)** UMAP projections of integrated data sets highlighting cluster genes used to identify neutrophils. **(G)** UMAP projections of integrated data sets highlighting cluster genes used to identify myeloid cells. **(H)** UMAP projections of integrated data sets highlighting cluster genes used to identify lymphocytes.

FIGURE S8

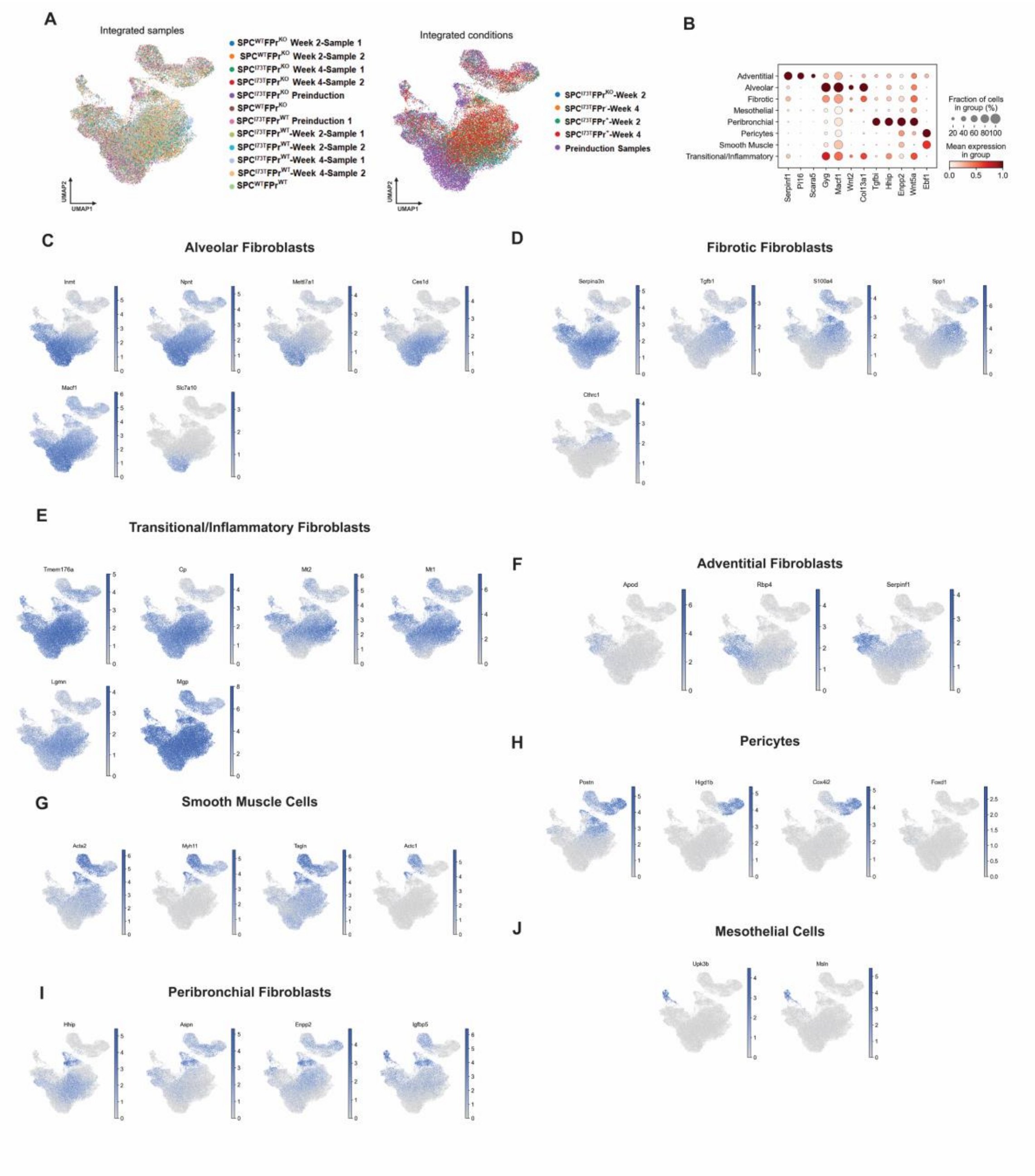

Supplemental Figure S8. Mesenchymal Cell Cluster Identification in *Sftpc^{I73T}* Integrated Single Cell Data Set. (A) UMAP projection of integrated mesenchymal compartment with color designations for each timepoint and genotype. **(B)** Alternative Marker genes for mesenchymal subsets graphed as a gradient dot plot. **(C)** UMAP projections of integrated mesenchyme highlighting cluster genes used to identify alveolar fibroblasts. **(D)** UMAP projections of integrated mesenchyme highlighting cluster genes used to identify fibrotic fibroblasts. **(E)** UMAP projections of integrated mesenchyme highlighting cluster genes used to identify transitional/inflammatory fibroblasts. **(F)** UMAP projections of integrated mesenchyme highlighting cluster genes used to identify adventitial fibroblasts. **(G)** UMAP projections of integrated mesenchyme highlighting cluster genes used to identify smooth muscle cells. **(H)** UMAP projections of integrated mesenchyme highlighting cluster genes used to identify pericytes. **(I)** UMAP projections of integrated mesenchyme highlighting cluster genes used to identify peribronchial fibroblasts. **(J)** UMAP projections of integrated mesenchyme highlighting cluster genes used to identify mesothelial cells.

FIGURE S9

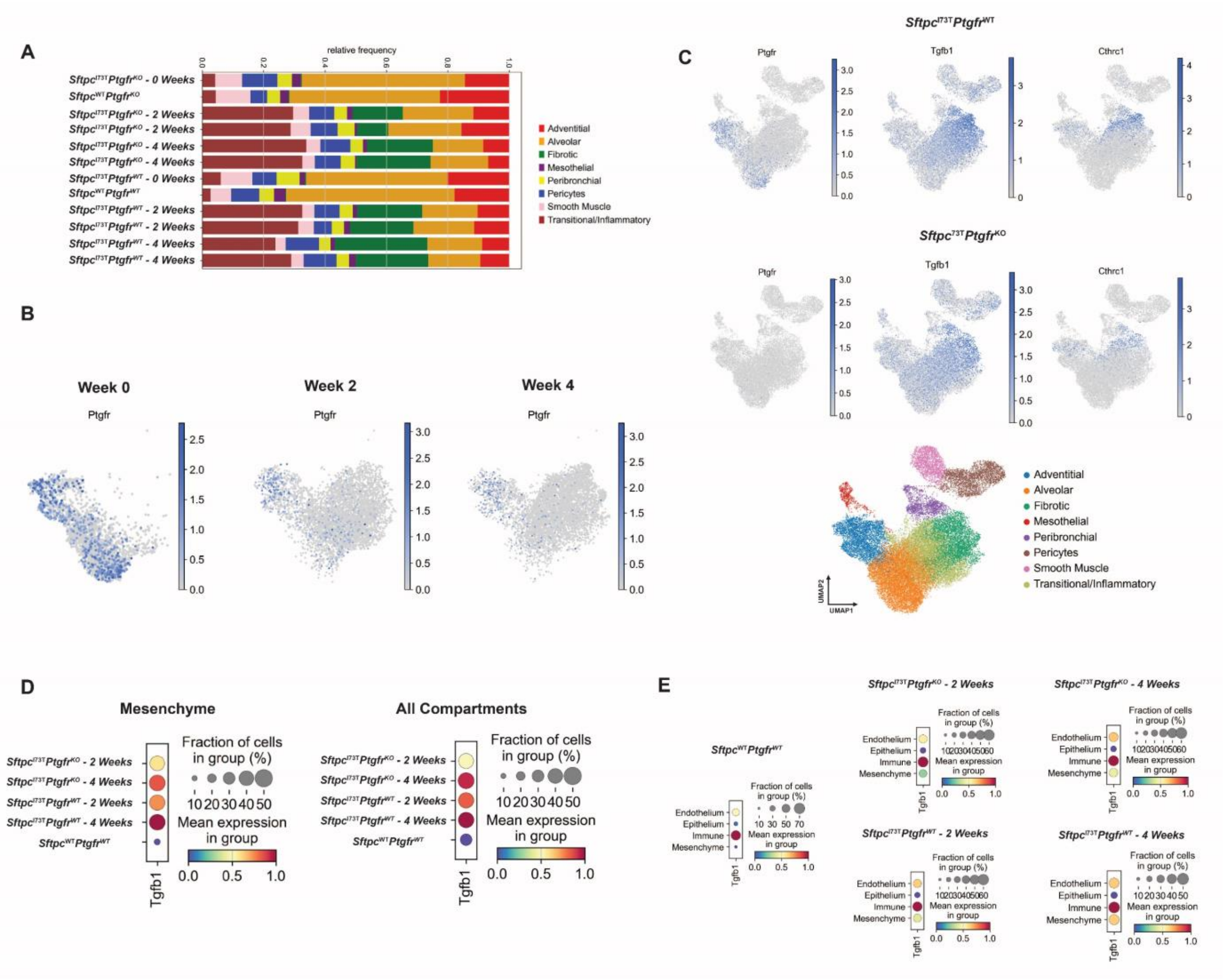

Supplemental Figure S9. (A) Frequency plot of mesenchymal subsets within individual samples. **(B)** UMAP projections of *Pdgfra* positive mesenchymal clusters divided by timepoint and highlighting the expression of *Ptgfr*. **(C)** UMAP projection of integrated mesenchymal clusters divided by genotype and highlighting profibrotic markers (D) *Tgfb1* expression in mesenchyme and all lung compartments combined (mesenchyme, endothelial, epithelial, and immune) expressed as dot plots across conditions and timepoint. (E) *Tgfb1* expression in each major lung compartment throughout the time course of the model expressed as dot plots and split by genotype and timepoint.

FIGURE S10

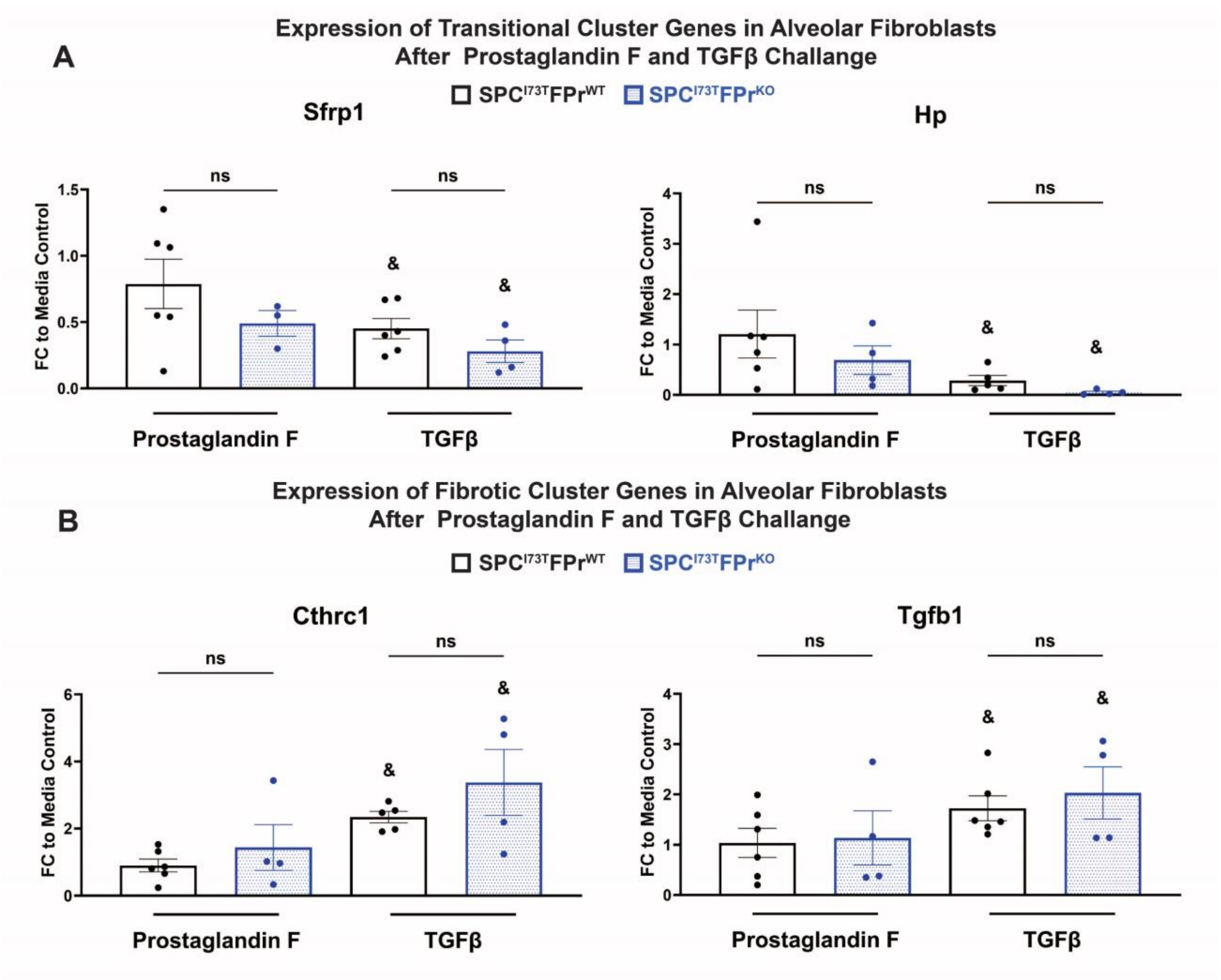

Supplemental Figure S10. (A) Quantification of transitional cluster maker genes after 48-hour challenge in alveolar fibroblasts demonstrates that Fp (500 nM) does not induce the transitional state in alveolar fibroblasts. While TGFβ (10ng/ml) decreases expression of transitional state marker genes independent of *Ptgfr* status (B) Quantification of fibrotic cluster marker genes after 48-hour challenge confirms that TGFβ promotes entry of alveolar fibroblasts into the fibrotic state independent of FPr status. Ordinary one-way ANOVA statistical testing was performed with significance between groups denoted by * : *p < 0.05 ** p<0.005 *** p<0.0005. Statistical significance between treatment and media control denoted by & : & p< 0.05

Supplemental Table 1: Antibodies for Flow Cytometry			
**Antibody**	**Clone**	**Catalog Number**	**Manufacturer**
**CD45**	30F-11	103110	Biolegend
**Epcam**	G8.8	118231	Biolegend
**Pecam**	MEC13.3	102533	Biolegend
**Pdgfra**	APA5	135907	Biolegend
**SiglecF**	E50-2440	562757	BD Biosciences
**CD11b**	M1/70	101205	eBiosciences
**CD11c**	HL3	561022	Biolegend
**Ly6G**	1A8	561236	Biolegend
**CD64**	X54-5/7.1	139305	Biolegend
**CD43**	S11	143205	Biolegend
**Ly6C**	HK1.4	128033	Biolegend
**CD3e**	145-2C11	563565	BD Biosciences
**Sca1**	D7	108107	Biolegend
**Mcam**	ME-9F1	134713	Biolegend
**CD9**	KMC8	752985	BD Biosciences

Supplemental Table 2: Primer Sequences		
**Target**	**Forward**	**Reverse**
**Sfrp1**	CCTCTAAGCCCCAAGGTACA	GACTGGAAGGTGGGACACTC
**Hp**	TCCACGATGAGAGCCCTGG	CATCCATAGAGCCACCGATGA
**Ces1d**	CCCATTGCTGGTCTGGTTGC	TGCCTTCAGCGAGTGGATAG
**Inmt**	AGCCTGCAGAACCTCTACCA	TGCCACCTGCTTCTGTCTCC
**Slc7a10**	GCACCATCATCATCGGGAAC	AGCACTCCAGAGCAGTAGGA
**Ebf1**	TCTATGTGCGCCTCATCGAC	AGGGAGTAGCTGCATGTTCC
**18S**	CGGCTACCACATCCAAGGAA	GCTGGAATTACCGCGGCT
**Cthrc1**	ATCCCAGGTCGGGATGGATT	CCAATCCCTTCACAGAGTCCT
**Col14a1**	TGAAGCACCCACAGCCATAG	TCCAGGCACCATAACCGTTC
**Col13a1**	CTAAAGGGGAGATGGGCCTG	ATTATCGGGTTGGAGCGCAG
**Pi16**	AACTATGAGCCTCCGGGGAA	GTCACCCTTGGAGGCGAATC
**Col1a1**	Mm00801666_g1	
**Col2a1**	Mm00483888_m1	
**Ptgfr**	Mm00436055_m1	
**Hprt**	Mm01545399_m1	

Supplemental Table 3 Mesenchymal Population Frequencies									
Genotype	Time Point	Adventitial	Alveolar	Fibrotic	Mesothelial	Peribronchial	Pericytes	Smooth Muscle	Transitional
$Sftpc^{I73T}$ $Ptgfr^{KO}$	Week 0	0.144	0.533	0.003	0.029	0.047	0.116	0.088	0.041
$Sftpc^{WT}$ $Ptgfr^{KO}$	Week 0	0.226	0.490	0.004	0.026	0.042	0.055	0.113	0.043
$Sftpc^{WT}$ $Ptgfr^{WT}$	Week 0	0.203	0.460	0.003	0.017	0.075	0.079	0.104	0.059
$Sftpc^{I73T}$ $Ptgfr^{WT}$	Week 0	0.179	0.549	0.003	0.037	0.048	0.092	0.068	0.026
$Sftpc^{I73T}$ $Ptgfr^{KO}$	Week 2	0.117	0.230	0.163	0.018	0.042	0.082	0.052	0.295
$Sftpc^{I73T}$ $Ptgfr^{KO}$	Week 2	0.156	0.239	0.101	0.008	0.055	0.088	0.065	0.287
$Sftpc^{I73T}$ $Ptgfr^{WT}$	Week 2	0.103	0.180	0.213	0.013	0.043	0.082	0.040	0.325
$Sftpc^{I73T}$ $Ptgfr^{WT}$	Week 2	0.115	0.199	0.206	0.020	0.040	0.058	0.051	0.312
$Sftpc^{I73T}$ $Ptgfr^{KO}$	Week 4	0.085	0.165	0.214	0.013	0.042	0.097	0.046	0.338
$Sftpc^{I73T}$ $Ptgfr^{KO}$	Week 4	0.069	0.188	0.240	0.005	0.047	0.084	0.041	0.325
$Sftpc^{I73T}$ $Ptgfr^{WT}$	Week 4	0.089	0.178	0.301	0.014	0.038	0.109	0.033	0.238
$Sftpc^{I73T}$ $Ptgfr^{WT}$	Week 4	0.095	0.170	0.236	0.023	0.040	0.108	0.040	0.290
